# Estimating the Global Clinical Burden of *Plasmodium falciparum* Malaria in 2007

**DOI:** 10.1371/journal.pmed.1000290

**Published:** 2010-06-15

**Authors:** Simon I. Hay, Emelda A. Okiro, Peter W. Gething, Anand P. Patil, Andrew J. Tatem, Carlos A. Guerra, Robert W. Snow

**Affiliations:** 1 Spatial Ecology and Epidemiology Group, Department of Zoology, University of Oxford, Oxford, United Kingdom; 2 Malaria Public Health and Epidemiology Group, Centre for Geographic Medicine, KEMRI–University of Oxford–Wellcome Trust Research Programme, Nairobi, Kenya; 3 Centre for Tropical Medicine, Nuffield Department of Clinical Medicine, University of Oxford, Oxford, United Kingdom; 4 Department of Geography, University of Florida, Gainesville, Florida, United States of America; 5 Emerging Pathogens Institute, University of Florida, Gainesville, Florida, United States of America; Papua New Guinea Institute of Medical Research, Papua New Guinea

## Abstract

**Background:**

The epidemiology of malaria makes surveillance-based methods of estimating its disease burden problematic. Cartographic approaches have provided alternative malaria burden estimates, but there remains widespread misunderstanding about their derivation and fidelity. The aims of this study are to present a new cartographic technique and its application for deriving global clinical burden estimates of *Plasmodium falciparum* malaria for 2007, and to compare these estimates and their likely precision with those derived under existing surveillance-based approaches.

**Methods and Findings:**

In seven of the 87 countries endemic for *P. falciparum* malaria, the health reporting infrastructure was deemed sufficiently rigorous for case reports to be used verbatim. In the remaining countries, the mapped extent of unstable and stable *P. falciparum* malaria transmission was first determined. Estimates of the plausible incidence range of clinical cases were then calculated within the spatial limits of unstable transmission. A modelled relationship between clinical incidence and prevalence was used, together with new maps of *P. falciparum* malaria endemicity, to estimate incidence in areas of stable transmission, and geostatistical joint simulation was used to quantify uncertainty in these estimates at national, regional, and global scales.

Combining these estimates for all areas of transmission risk resulted in 451 million (95% credible interval 349–552 million) clinical cases of *P. falciparum* malaria in 2007. Almost all of this burden of morbidity occurred in areas of stable transmission. More than half of all estimated *P. falciparum* clinical cases and associated uncertainty occurred in India, Nigeria, the Democratic Republic of the Congo (DRC), and Myanmar (Burma), where 1.405 billion people are at risk.

Recent surveillance-based methods of burden estimation were then reviewed and discrepancies in national estimates explored. When these cartographically derived national estimates were ranked according to their relative uncertainty and replaced by surveillance-based estimates in the least certain half, 98% of the global clinical burden continued to be estimated by cartographic techniques.

**Conclusions and Significance:**

Cartographic approaches to burden estimation provide a globally consistent measure of malaria morbidity of known fidelity, and they represent the only plausible method in those malaria-endemic countries with nonfunctional national surveillance. Unacceptable uncertainty in the clinical burden of malaria in only four countries confounds our ability to evaluate needs and monitor progress toward international targets for malaria control at the global scale. National prevalence surveys in each nation would reduce this uncertainty profoundly. Opportunities for further reducing uncertainty in clinical burden estimates by hybridizing alternative burden estimation procedures are also evaluated.

*Please see later in the article for the Editors' Summary*

## Abstract 2

Simon Hay and colleagues derive contemporary estimates of the global clinical burden of *Plasmodium falciparum* malaria (the deadliest form of malaria) using cartography-based techniques.

## Editors' Summary

**Background:**

Malaria is a major global public-health problem. Nearly half the world's population is at risk of malaria, and *Plasmodium falciparum* malaria—the deadliest form of the disease—causes about one million deaths each year. Malaria is a parasitic disease that is transmitted to people through the bite of an infected mosquito. These insects inject a parasitic form known as sporozoites into people, where they replicate briefly inside liver cells. The liver cells then release merozoites (another parasitic form), which invade red blood cells. Here, the merozoites replicate rapidly before bursting out and infecting more red blood cells. This increase in the parasitic burden causes malaria's characteristic symptoms—debilitating and recurring fevers and chills. Infected red blood cells also release gametocytes, which infect mosquitoes when they take a blood meal. In the mosquito, the gametocytes multiply and develop into sporozoites, thus completing the parasite's life cycle. Malaria can be prevented by controlling the mosquitoes that spread the parasite and by avoiding mosquito bites. Effective treatment with antimalarial drugs also helps to reduce malaria transmission.

**Why Was This Study Done?:**

In 1998, the World Health Organization (WHO) and several other international agencies launched Roll Back Malaria, a global partnership that aims to provide a coordinated, global approach to fighting malaria. For this or any other malaria control initiative to be effective, however, an accurate picture of the global clinical burden of malaria (how many people become ill because of malaria and where they live) is needed so that resources can be concentrated where they will have the most impact. Estimates of the global burden of many infectious diseases are obtained using data collected by national surveillance systems. Unfortunately, this approach does not work very well for malaria because in places where malaria is endemic (always present), diagnosis is often inaccurate and national reporting is incomplete. In this study, therefore, the researchers use an alternative, “cartographic” method for estimating the global clinical burden of *P. falciparum* malaria.

**What Did the Researchers Do and Find?:**

The researchers identified seven *P. falciparum* malaria-endemic countries that had sufficiently reliable health information systems to determine the national clinical malaria burden in 2007 directly. They divided the other 80 malaria endemic countries into countries with a low risk of transmission (unstable transmission) and countries with a moderate or high risk of transmission (stable transmission). In countries with unstable transmission, the researchers assumed a uniform annual clinical incidence rate of 0.1 cases per 1,000 people and multiplied this by population sizes to get disease burden estimates. In countries with stable transmission, they used a modeled relationship between clinical incidence (number of new cases in a population per year) and prevalence (the proportion of a population infected with malaria parasites) and a global malaria endemicity map (a map that indicates the risk of malaria infection in different countries) to estimate malaria incidences. Finally, they used a technique called “joint simulation” to quantify the uncertainty in these estimates. Together, these disease burden estimates gave an estimated global burden of 451 million clinical cases of *P. falciparum* in 2007. Most of these cases occurred in areas of stable transmission and more than half occurred in India, Nigeria, the Democratic Republic of the Congo, and Myanmar. Importantly, these four nations alone contributed nearly half of the uncertainty in the global incidence estimates.

**What Do These Findings Mean?:**

These findings are extremely valuable because they provide a global map of malaria cases that should facilitate the implementation and evaluation of malaria control programs. However, the estimate of the global clinical burden of *P. falciparum* malaria reported here is higher than the WHO estimate of 247 million cases each year that was obtained using surveillance-based methods. The discrepancy between the estimates obtained using the cartographic and the surveillance-based approach is particularly marked for India. The researchers discuss possible reasons for these discrepancies and suggest improvements that could be made to both methods to increase the validity and precision of estimates. Finally, they note that improvements in the national prevalence surveys in India, Nigeria, the Democratic Republic of the Congo, and Myanmar would greatly reduce the uncertainty associated with their estimate of the global clinical burden of malaria, an observation that should encourage efforts to improve malaria surveillance in these countries.

**Additional Information:**

Please access these Web sites via the online version of this summary at http://dx.doi.org/10.1371/journal.pmed.1000261.

- A *PLoS Medicine* Health in Action article by Hay and colleagues, a Research Article by Guerra and colleagues, and a Research Article by Hay and colleagues provide further details about the global mapping of malaria risk
- Additional national and regional level maps and more information on the global mapping of malaria are available at the Malaria Atlas Project
- Information is available from the World Health Organization on malaria (in several languages)
- The US Centers for Disease Control and Prevention provide information on malaria (in English and Spanish)
- Information is available from the Roll Back Malaria Partnership on its approach to the global control of malaria (in English and French)
- MedlinePlus provides links to additional information on malaria (in English and Spanish)

## Introduction

Estimating the disease burden posed by malaria is an important public health challenge [1]–[9]. The clinical consequences of *Plasmodium falciparum* infection have several features that confound traditional approaches to disease burden and disability measurement [10],[11]. First, not all infections result in progression to disease, notably in areas of stable transmission [12], where populations have acquired clinical immunity [13]. The overall risk of clinical disease has a curvilinear and uncertain association with the risk of infection as a combined function of age at first infection and immunity [13]–[18]. Second, the dominant symptom of fever, or other symptoms, does not distinguish malaria from other locally prevalent infections [19]–[23]. As a consequence, the routine reporting of “malaria” can overestimate disease rates by assuming that most fevers are malaria [24],[25] and that fevers associated with an infection are causally linked to that infection [20],[26]. Third, with few exceptions across malaria-endemic countries, fevers or other malaria-like syndromes are often self-medicated and may resolve regardless of cause before reaching formal health systems [27]. Fourth, inaccurate diagnoses [21],[25],[28] might be used to report disease rates, and these errors may be compounded through inadequate and incomplete national reporting systems [29]–[38].

To circumvent some of the clinical, treatment, and reporting problems inherent in malaria burden estimation, we previously computed the global incidence of *P. falciparum* clinical disease [5] for 2002, using assemblies of epidemiological data and a modified categorical map of historical malaria endemicity [39]. The publication of (i) the revised global spatial limits of *P. falciparum* transmission [40], (ii) a contemporary geostatistical description of *P. falciparum* malaria endemicity within these limits [41], and (iii) updates of the modelled relationship between clinical incidence and prevalence [42] have resulted in a substantially improved evidence base from which to revisit estimates of the clinical burden of *P. falciparum*, defined as the primary acute clinical event resulting from malaria infection at all ages. Most significantly, a geostatistical space–time joint simulation framework [43] is combined with these improved cartographic and epidemiological data sources to quantify uncertainty in the mapped outputs and to propagate it appropriately into the derived burden estimates. Using these joint simulation procedures we have built upon previous approaches to produce the first continuous map of global clinical *P. falciparum* incidence, and we use this to estimate the global clinical burden of *P. falciparum* malaria in 2007. These estimates are then compared with those available from surveillance, and the opportunity for the further hybridization of these techniques is discussed.

## Methods

### Analysis Outline

**Figure 1:**
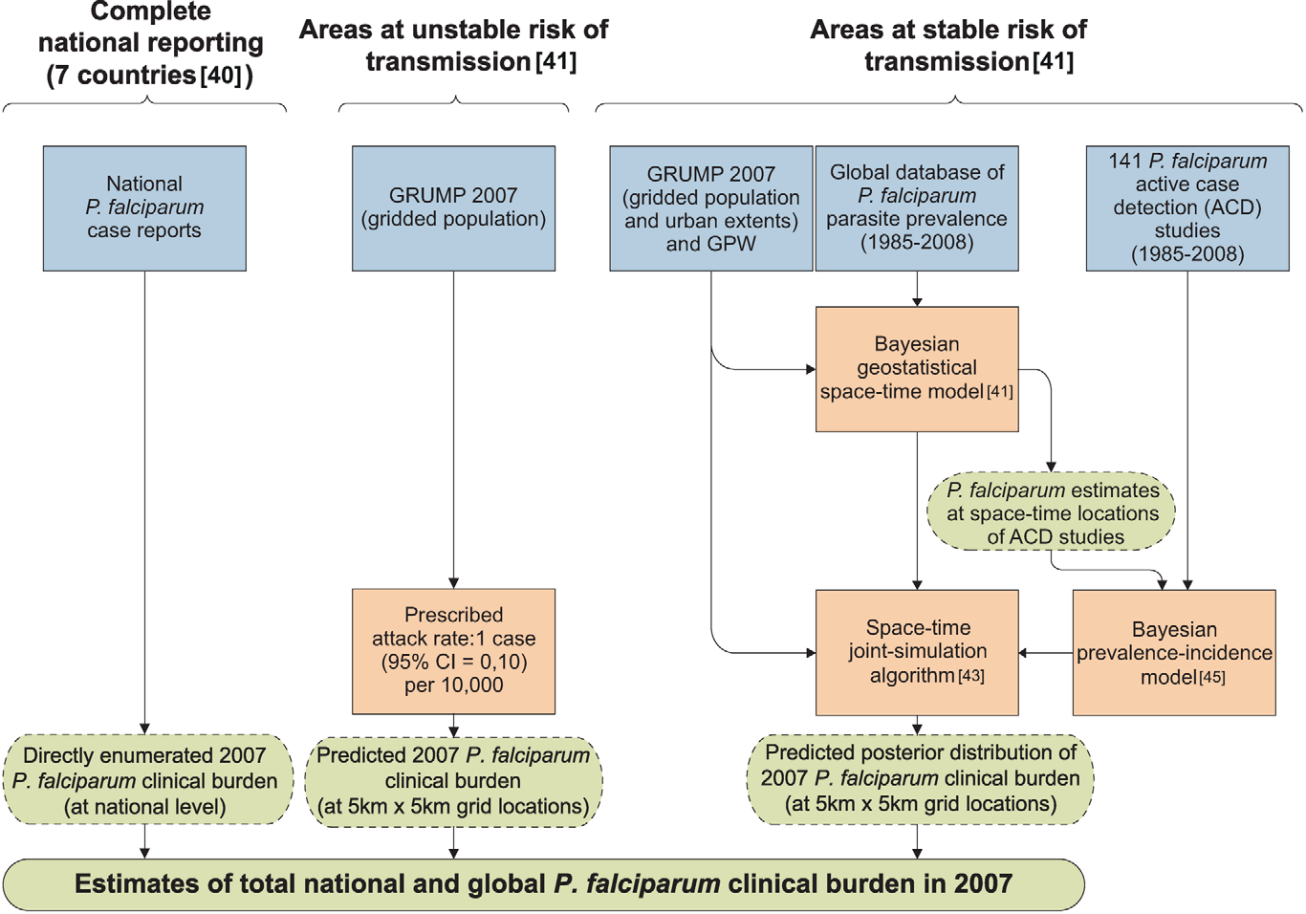
Schematic diagram showing the procedure for burden estimation. Blue boxes describe input data, orange boxes models and experimental procedures, dashed green rods intermediate output, and solid green rods the final output. The seven countries with reliable national reporting were Belize, Iran, Kyrgyzstan, Panama, Saudi Arabia, South Africa, and Tajikistan. The areas of unstable and stable transmission are defined as having less or more than one case per 10,000 PA, respectively [40],[41].

**Figure 2:**
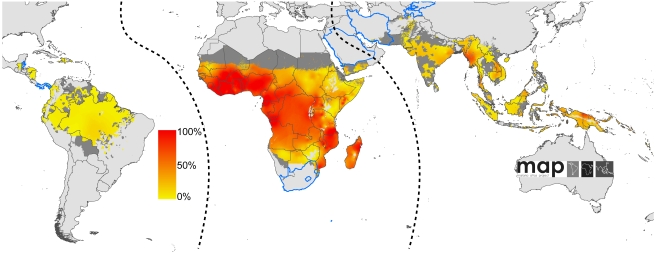
Global limits and endemicity of *P. falciparum* in 2007. The land area was defined as no risk (light grey), unstable risk (medium grey areas, where *Pf*API <0.1‰ PA), and stable risk (where *Pf*API >0.1‰ PA) [40] with endemicity (*Pf*PR in the 2- up to 10-year age group, *Pf*PR_2–10_) displayed as a continuum of yellow to red between 0% and 100%. The dashed lines separate the Americas, Africa+, and the CSE Asia region, respectively, from left to right. The seven countries with thick blue borders have very low *P. falciparum* burden and reliable national health information systems.

**Figure 3:**
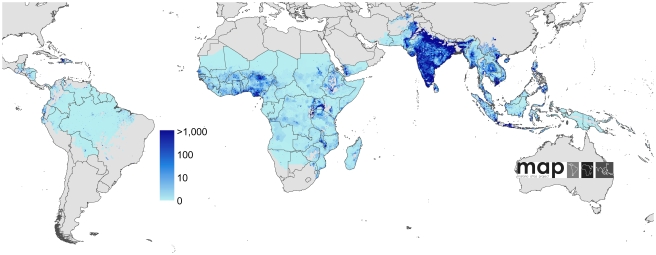
Global human population density in 2007. Human population density [44] in persons per km^2^ is displayed on a logarithmic colour scale within the limits of *P. falciparum* transmission. No malaria risk is shown in light grey.

**Figure 4:**
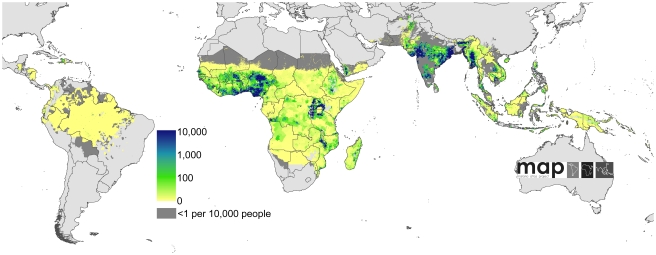
Global clinical burden of *P. falciparum* in 2007. Bayesian geostatistical estimates (posterior means) of the number of all-age clinical cases per 5×5 km pixel displayed on a logarithmic colour scale between 0 and 10,000 cases, within the stable limits of *P. falciparum* transmission. Dark and light grey areas are as described in Figure 2.

**Figure 5:**
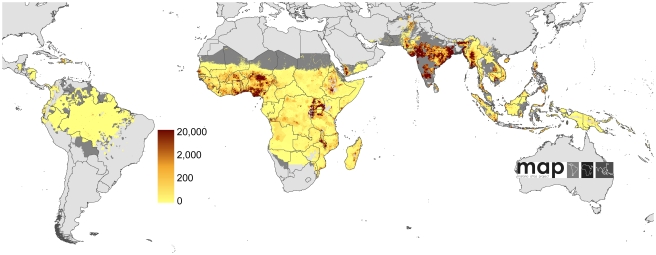
Uncertainty in the global clinical burden of *P. falciparum* in 2007. Bayesian geostatistical model-based prediction uncertainty (posterior standard deviations) on a logarithmic colour scale between 0 and 20,000 cases, within the stable limits of *P. falciparum* transmission. No model-based uncertainty metrics were produced for areas of unstable transmission. Dark and light grey areas are as described in Figure 2.

A schematic overview of the analysis procedures is provided in Figure 1. In brief, of the 87 countries classified as endemic for *P. falciparum* malaria [40], seven had sufficiently reliable health information systems for case report data to be used directly to enumerate clinical burden for 2007. We divided the population at risk (PAR) in the remaining 80 countries into regions of unstable and stable risk of transmission [40] (Figure 2). In unstable regions, a uniform clinical incidence rate was adopted of 0.1 case per 1,000 per annum (PA). This rate was multiplied by a population surface [44] for 2007 (Figure 3) and aggregated to obtain country and regional case estimates for these unstable areas. Upper and lower bounds were defined using uniform rates of zero and one case, respectively, per 1,000 PA. In stable regions, we used a previously defined Bayesian geostatistical model that took an assembly of space–time distributed *P. falciparum* parasite rate (*Pf*PR) surveys and generated realisations of continuous age-standardized prevalence within the limits of stable transmission [41]. We then used a Bayesian nonparametric model [42] of a collection of all-age active case detection studies, to describe the uncertain relationship between the clinical incidence rate and the underlying age-standardized parasite prevalence. These two models were integrated in a geostatistical space–time joint simulation framework to generate joint realisations of clinical attack rate for every pixel as a function of the predicted underlying prevalence [43] (Protocol S1). These attack rates were then multiplied by the corresponding pixel population totals to yield joint realisations of a clinical burden surface (Figures 4 and 5). This joint simulation framework supported the aggregation of per-pixel burden estimates into defined spatial units, whilst preserving a space–time uncertainty structure, allowing country and regional estimates of burden to be made with appropriate credible intervals (Table 1, Protocol S2). Each of these analytical components are now discussed in more detail.

**Table 1:** Numbers of *Plasmodium falciparum* clinical attacks by region globally in 2007.

Category	Americas (16 countries)	Africa+ (47 countries)	CSE Asia (19 countries)	Total
Reliable reporting (cases^a^)	32 (Panama, Belize)	2,717^b^ (Saudi Arabia, South Africa)	618 (Kyrgyzstan, Tajikistan, Iran)	3,367
Unstable risk^c^ (cases^a^)	5,455 (0–54,550)	1,892 (0–18,920)	98,049 (0–980,490)	105,395 (0–1,053,950)
Stable risk^c^ (millions of cases^a^)	3.04 (1.17–6.70)	270.88 (241.13–300.56)	176.90 (89.21–269.58)	450.83 (348.76–552.22)
Total (millions of cases^a^)	3.05 (1.17–6.76)	270.89 (241.13–300.58)	177.00 (89.21–270.56)	450.93 (348.76–553.27)

The regional groupings are illustrated in Figure 1.

a Case numbers from countries with reliable reporting and areas of unstable risk are presented directly whilst those from areas of stable risk are presented in millions of cases, rounded to the nearest 10,000, reflecting the larger numbers and lower precision associated with these model-based estimates.

b Presumed to be all *P. falciparum*, although autochthonous case reports did not specify.

c Excluding countries with reliable case data.

### Defining Populations and Global Regions

The Global Rural Urban Mapping Project (GRUMP) alpha version [44] provides gridded population counts and population density estimates for the years 1990, 1995, and 2000, adjusted to the United Nations' national population estimates. Population counts for the year 2000 were projected to 2007 by applying national, medium variant, intercensal growth rates [45] by country using methods previously described [46] (Figure 3).

We have modified the World Health Organization (WHO) regional country groupings, recognizing that these geopolitical boundaries do not conform to the biogeographical determinants of malaria risk and thus disease burden [41],[47],[48]. For the purposes of disease risk estimation we have used three malaria regional groupings: Africa+ (including Yemen and Saudi Arabia, which share the same dominant *Anopheles* vectors as mainland Africa [49]), the Americas, and the combined regions of Near East, Asia, and the Pacific that we refer to as Central and South East (CSE) Asia (Figure 2). To facilitate comparison with other estimates, however, we have also shown the results aggregated by the regional groupings of the WHO (Protocol S2).

### Defining the Limits of Stable and Unstable *P. falciparum* Transmission

To define the global spatial limits of *P. falciparum* transmission, we previously assembled confirmed *P. falciparum* clinical case data for 41 *P. falciparum* malaria-endemic countries (*Pf*MECs) outside of Africa [40]. National case reported data were expressed as *P. falciparum* annual parasite incidence (*Pf*API) derived from various combinations of active case detection (fever surveys in communities where every person presenting with a fever is tested for parasite infection) and passive case detection (reports from febrile patients attending the local health services) and usually expressed together as the number infected per 1,000 PA [50]–[52]. These data were provided by malaria coordinating officers in the WHO regional offices of the Eastern Mediterranean (EMRO), Europe (EURO), South East Asia (SEARO), and the Western Pacific (WPRO) at the highest available administrative level unit between 2002 and 2007. Among the countries in the American Regional Office (AMRO), *Pf*API data from national surveillance systems in Brazil, Colombia, Peru, and Honduras were obtained directly from personal communication with national malaria specialists.

The *Pf*API data were mapped to first, second, or third administrative level units and used to classify areas as no risk (zero cases) and either unstable or stable risk if the number of confirmed cases was lower or higher than 0.1 case per 1,000 PA, respectively [40]. The unstable/stable classification was based on a review of the statistical, logistical, and programmatic reasons underpinning the *Pf*API levels used to define phases and action points during the Global Malaria Eradication Program [12],[53]–[55]. In addition, no transmission was assumed where medical intelligence from international travel advisories or national malaria control programmes stated no malaria risk or where the temperature was too low for sporogony to complete within the average lifespan of the local dominant vector species [49]. Measures of aridity were used to define areas in which transmission is biologically plausible in isolated manmade breeding sites, but overall transmission in surrounding areas is limited by its effects on anopheline survival, and the clinical incidence is likely to be less than 0.1 case per 1,000 PA. The spatial extents of stable and unstable risk defined using these inputs are shown (Figure 2).

### Defining *P. falciparum* Clinical Incidence in Areas of Reliable Case Detection

Paradoxically, where the incidence of clinical malaria events are rare, their rapid detection and notification becomes increasingly important as part of national malaria control strategies, demanding more sophisticated surveillance [51],[55]–[57]. This is particularly true for countries aiming to attain or maintain WHO accredited elimination status [58]–[60]. Of the 87 *Pf*MECs, we have identified seven countries that are relatively wealthy and have specified a goal of *P. falciparum* elimination where case-detection systems are an integral part of the control strategies [58]–[60]: Panama, Belize, Tajikistan, Kyrgyzstan, Iran, Saudi Arabia, and South Africa (Figure 2). For these seven countries, we have used the national reports for 2007 of all notified, locally acquired infections submitted to regional WHO offices (see Acknowledgments) as the definitive estimate of case burden. These countries are characterised by having a small number of annual cases, with a large proportion of the population living in areas of no risk or unstable transmission and are therefore likely to represent a very small proportion of the global *P. falciparum* malaria burden [40].

### Defining Malaria Incidence in Areas of Unstable *P. falciparum* Malaria Transmission

We estimate that almost one billion people were living in areas where *P. falciparum* transmission was unstable in 2007 [40] (Figure 2). Defining annualized disease risk in these areas from empirical data is difficult, as epidemiological investigations for research or survey purposes are rare. Nevertheless, in computing disease burdens it is important to impute some measure of completeness of formal malaria reporting within these marginal, unstable transmission areas. A number of malaria treatment-seeking behaviour studies and qualitative examinations of routine malaria reporting frequency suggest large inadequacies in a range of national reporting systems from a variety of causes that can act multiplicatively: Cambodia (actual number of cases 2.7× greater than reported) [35], India (9–50×) [28],[61]–[65], Mozambique (2.7×) [32], Pakistan (5.9×) [30], Peru (4.3×) [34], Solomon Islands (4.7×) [38], Sri Lanka (1.9×) [29], and Syria (4.5×) [31].

There are remarkably few specific investigations of the completeness of malaria case notification systems in different settings. Only four reports provide an estimate of the numbers of cases likely to be missed by routine health system surveillance compared to more aggressive, active case detection methods in the same communities over the same time period. In the Yanomami area of Brazil, approximately 1.25 more events were detected by active detection than were reported to the routine health system [57]. Across different years at different sites the ratio of active to routine, passive detection varied from 4.5 to 42.1 in Vietnam [66], with similar under-reporting rates documented in Cambodia [67]. A 5-fold difference in survey-to-passive rates of case detection has been reported in Yunnan Province in China [68]. It is not possible to provide an evidence-based under-reporting correction factor that is specific for every national malaria information system. We have therefore elected to use a single worst-case rate of 10-fold under-reporting across all countries. We hence assume for all unstable areas a uniform incidence of 0.1 case per 1,000 PA, with a lower confidence bound of zero and an upper confidence bound assuming a 10-fold under-reporting rate; equating to one case per 1,000 PA.

### Defining Malaria Incidence in Stable Endemic Areas

We estimated that in 2007, approximately 1.4 billion people lived in areas of stable *P. falciparum* transmission [40] (Figure 2). In these areas, we considered that case-reporting through routine health information systems was too unreliable for the calculation of incidence due to inadequate reporting coverage (see above), widespread self-medication [27], and poor diagnosis [21],[25]. Instead, we developed a model-based cartographic method for deriving estimates in the areas of stable transmission in which clinical incidence was modelled as a function of the underlying endemicity (parasite prevalence). This procedure required: (i) a spatially continuous model for endemicity; (ii) a further model to predict incidence as a function of endemicity; (iii) reliable data on 2007 population distribution; and (iv) a technique for combining these components so that the uncertainty inherent in the component models was propagated into the resulting burden estimates. These components are now outlined in turn, with additional statistical details provided in Protocol S1.

To estimate stable transmission intensity, a Bayesian space-time geostatistical modelling framework was developed to interpolate empirical estimates of age-corrected parasite prevalence derived from 7,953 community surveys undertaken between 1985 and 2008 across 83 malaria-endemic countries. This model has been described in detail elsewhere [41] and its output allows for a continuous, urban-adjusted, contemporary estimate of parasite prevalence in children aged from 2 up to 10 years (*Pf*PR_2–10_) at a pixel spatial resolution of 5×5 km for the year 2007 (Figure 2).

**Figure 6:**
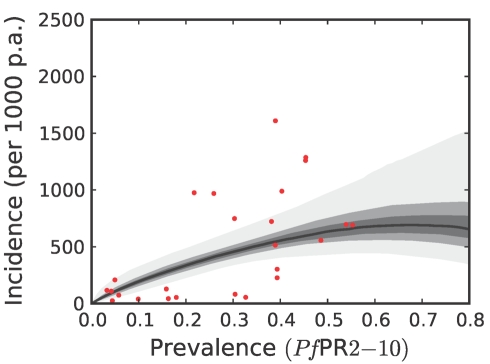
The posterior distribution of the prevalence-incidence relationship (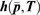, see Methods) in the Africa+ region. The relationship is plotted between malaria endemicity (*Pf*PR in the 2- up to 10-year age group, *Pf*PR_2–10_) and all-age incidence (clinical cases per thousand of the population PA) [42]. Please see reference [42] for a full description of the data, methods, and techniques used to define this relationship. The light grey, medium grey and dark grey regions define the 95%, 50%, and 25% credible intervals, respectively. The solid black line is the median and the data are shown as red dots.

**Figure 7:**
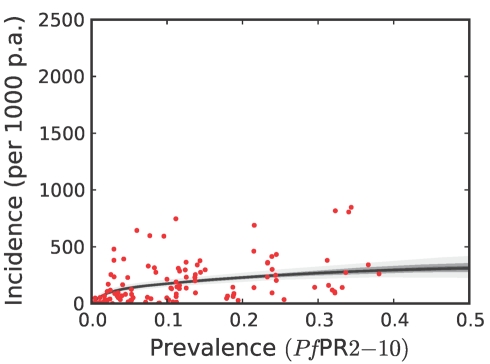
The posterior distribution of the prevalence-incidence relationship (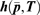, see Methods) in the combined CSE Asia region and the Americas. The techniques and colours used are identical to Figure 6.

**Figure 8:**
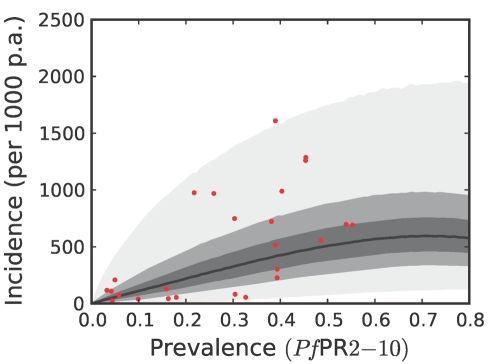
The predictive distribution of the incidence that would actually be observed by weekly surveillance over a two-year period in the Africa+ region. Please see reference [42] for a full description of the data, methods, and techniques used to define this relationship. The light grey, medium grey, and dark grey regions define the 95%, 50%, and 25% credible intervals, respectively. The solid black line is the median and the data are shown as red dots. Note that the data points were collected using different surveillance intervals over different time periods, and therefore should not be expected to follow the distribution predicted by the model exactly. The observed incidences are included in the figure as a visual aid only.

**Figure 9:**
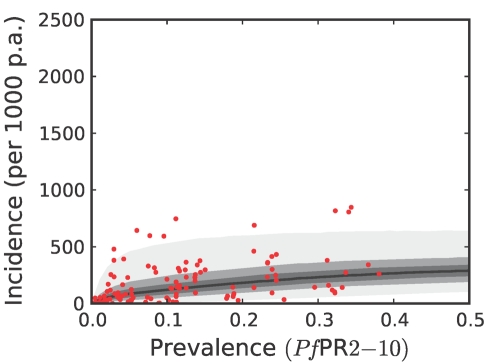
The predictive distribution of the incidence that would actually be observed by weekly surveillance over a two-year period in the combined CSE Asia region and the Americas. The techniques and colours used are identical to Figure 8.

To estimate clinical incidence, formal literature searches were conducted for *P. falciparum* malaria incidence surveys undertaken prospectively through active case detection at least every 14 days [42]. The incidence surveys were time–space matched with estimates of parasite prevalence derived from the geostatistical model described above [41]. Potential relationships between all-age clinical incidence and age-standardized parasite prevalence were then specified in a nonparametric Gaussian process model with minimal, biologically informed, prior constraints. A temporal volatility model was incorporated to describe the variance in the observed data and Bayesian inference was used to choose between the candidate models [42]. Separate relationships were preferred for each of the three regions defined globally (Figure 2) to accommodate regional-specific differences in the dominant vector species [47],[49],[69], the impact of drug resistance on recrudescent clinical attacks [70], the possible modification of *P. falciparum* clinical outcomes in areas of *P. vivax* co-infection [71],[72], and the genetic contribution to disease risk of inherited haemoglobin disorders [73]. Due to the sparse data in the Americas, however, this region was combined with CSE Asia. In the Africa+ region and the combined Americas and CSE Asia region, clinical incidence increased slowly and smoothly as a function of infection prevalence (Figures 6, 7, 8, and 9). In the Africa+ region, when infection prevalence exceeded 40%, clinical incidence reached a maximum of 500 cases per 1,000 PA (Figure 6). In the combined Americas and CSE Asia regions this maximum was reached at 250 cases per 1,000 PA (Figure 7).

Both the geostatistical endemicity and the endemicity–incidence models were specified in a fully Bayesian framework. The output of the former was a large set of realisations (*n* = 250,000): possible maps that, together, represented the modelled uncertainty in endemicity at each location. Similarly, the output of the endemicity–incidence model was a large set (*n* = 250,000) of possible forms of the endemicity-incidence curve that encompassed the modelled uncertainty in this relationship (Figures 6, 7, 8, and 9). To combine the uncertainty from both models, each realisation of the uncertainty map was used as input into a realisation of the endemicity–incidence model to obtain a realisation of a 5×5 km resolution incidence map. This was downscaled to 1×1 km resolution and multiplied with the 2007 population surface to obtain, for every grid square, a realisation of the number of clinical cases in 2007. By repeating this procedure for every model realisation, a set of 250,000 burden values was generated for every grid square, approximating a complete posterior distribution for the estimates. Because each realisation of the endemicity map was jointly simulated, rather than calculated on a pixel-by-pixel basis, each realisation of burden could be aggregated spatially or temporally, whilst maintaining the correct variance structure. This allowed burden realisations at each pixel to be combined spatially to generate estimates of national and regional burdens with appropriate credible intervals. Joint simulation at this scale is enormously computationally intensive and a bespoke algorithm was developed to implement this stage of the analysis. The algorithm is presented elsewhere [43] and the statistical details are summarised in Protocol S1.

## Results

The combined clinical burden of the seven nations with comprehensive reporting was 3,367 cases in 2007 (Table 1, Protocol S2). Multiplying the population surface (Figure 3) by the assumed incidence rate in unstable areas (see Methods) produced an estimate of 105,395 clinical cases of *P. falciparum* malaria in areas of unstable transmission (Table 1, Protocol S2), with a plausible range between zero and 1,053,950. The modelling procedures in the stable areas generated an estimate of 451 million cases (lower 95% credible interval 349 million and upper 95% credible interval 552 million) of *P. falciparum* malaria in areas of stable transmission in 2007, of which 271 (241–301) million were estimated to have occurred in the Africa+ region, 177 (89–270) million in the CSE Asia region and 3 (1–7) million in the Americas (Table 1).

**Figure 10:**
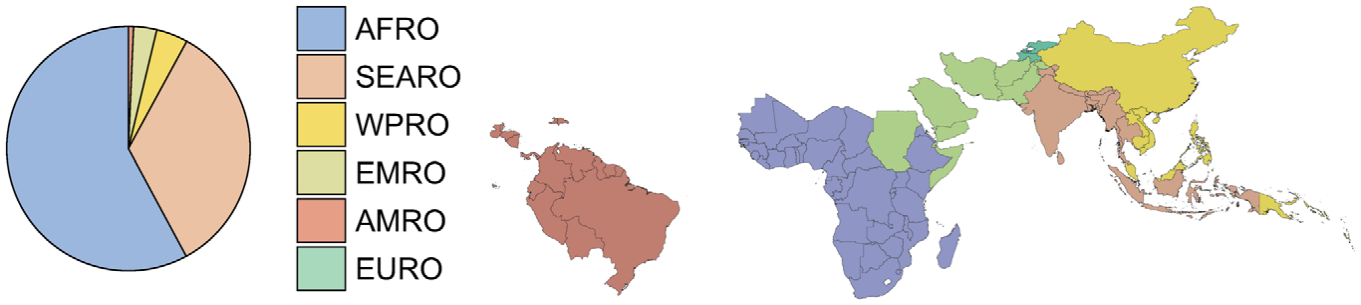
Pie chart of *P. falciparum* clinical cases in 2007. The pie chart shows the fraction of the 451 million cases of total clinical burden in each of the World Health Organization regions (Protocol S2). In the pie the regions are ordered counterclockwise starting at the top, from highest to lowest burden. The plotted area representing the EURO region is too thin to be visible. The thumbnail map shows the country composition of the WHO regions for all 87 *P. falciparum* endemic countries.

Combining our estimates from the seven countries with comprehensive case reporting with those from areas of unstable and stable transmission in the remaining 80 *Pf*MECs, we estimate that in 2007 there were 451 (349–553) million clinical cases of *P. falciparum* malaria. A continuous map of these incidence predictions is provided (Figure 4), with an additional map of the pixel-specific uncertainty (Figure 5). In addition to the regional summaries presented (Table 1), estimates of clinical burden are summarized for each country and for each of the WHO global regions (Figure 10 and Protocol S2). It is notable that more than half (51%) of the world's estimated *P. falciparum* clinical cases derive from just four countries: India, Nigeria, DRC, and Myanmar (Burma) (Figure 4 and Protocol S2) and that, in addition, these nations contribute 48% of the uncertainty (Figure 5) in the global incidence estimates.

Regional summary estimates of *P. falciparum* malaria cases in unstable and stable transmission areas are summarized in Table 1 and are also shown for the WHO regions in Figure 10. It is clear that African populations suffered the largest proportion (60%) of the 451 million clinical cases of *P. falciparum* estimated globally in 2007 (Figure 10, Table 1 and Protocol S2). The highest-burden countries in Africa are Nigeria and DRC, both countries with extensive regions of high endemicity (Figure 2) and large populations (Figure 3). These two countries account for 23% of the world's *P. falciparum* disease burden (Protocol S2). Less than 1% of the global *P. falciparum* burden occurred in the Americas, where transmission intensity is almost universally low or unstable (Figure 2). We estimate that the remaining 39% of global burden in 2007 occurred in the CSE Asia region (Table 1). In this region, the immense population living at risk of *P. falciparum* malaria means that, despite a low prevalence [41] (Figure 2) and the lower endemicity–incidence relationship [42] (Figure 7), cases in CSE Asia add substantially to the global disease burden (Table 1). At a country level, India and Myanmar contribute 22.6% and 5.8%, respectively, of the total number of clinical cases due to *P. falciparum* worldwide (Protocol S2).

## Discussion

We have used a combination of methods, including a joint simulation of incidence in areas of stable transmission, to estimate 451 (349–552) million clinical cases of *P. falciparum* malaria in 2007: 3 (1–7) million in the Americas, 271 (241–301) in the Africa+ region, and 177 (89–270) in the CSE Asia region.

### Morbidity in Areas of Unstable Transmission

We have accepted as accurate the surveillance reports of seven relatively high income and low burden *Pf*MECs, all nations with credible plans for malaria elimination [59],[60],[74]–[76]. We have further attempted to describe clinical disease incidence in areas of the world that we classify as unstable risk [40], which were home to almost a billion people in 2007. We know relatively little about the epidemiology of *P. falciparum* in the 40% of the global PAR of *P. falciparum* malaria living in unstable transmission areas. These areas are notoriously difficult to define in terms of potential disease outcomes; they may go several years without a single autochthonous case, transmission is extremely focal and, importantly, investigation of the clinical epidemiology is prohibitively expensive because of the rarity of the disease [77]. We have, therefore, defaulted to national reporting systems as an entry point to the definition of risk and have used surveys of under-reporting rates to define plausible ranges of the disease burden in these marginal transmission zones. We estimate that there were 105,395 (0–1,053,950) cases of *P. falciparum* in unstable transmission areas in 2007. Despite being relatively crudely defined, these sums represent only 0.02% of the global clinical *P. falciparum* burden. Therefore, while these cases are of significant concern to those nations with large populations at unstable risk and to those considering elimination [59],[60],[74]–[76], they make a very small contribution to the estimation of the global *P. falciparum* burden.

### Morbidity in Stable Areas

We have improved upon a *P. falciparum* disease burden estimation rubric that has been used several times previously for Africa [1],[3],[4],[6],[7] and once before globally [5]. This method requires an understanding of the basic clinical epidemiology of *P. falciparum* malaria, its relationship to transmission intensity and the use of empirical, longitudinal observations in populations exposed to different conditions of transmission. However, these empirical studies of clinical incidence are not without their own caveats [42]. Longitudinal surveillance over a complete annual malaria transmission cycle within the same cohort is likely to underestimate the “natural” risk of disease given the ethical need to treat effectively all detected infections or clinical events. These studies are also conducted throughout a range of region-specific co-species infection [78], HIV/AIDS prevalence [79], and drug resistance [80] conditions. The number of studies meeting our inclusion criteria remains low, so these covariate determinants of clinical risk cannot be adequately modelled or controlled for in this series [42]. We have considered all infections that are associated with a reported or measured febrile event as clinical malaria. This seems appropriate under conditions of low transmission intensity, but as transmission intensity increases, the proportion of fevers that can be causally linked to malaria infection declines [26],[81]. Consequently, our estimates of clinical attack rates at the highest levels of transmission are likely to be overestimates of true *P. falciparum* clinical incidence. Locally derived age- and transmission-dependent aetiological fraction estimates were not available for the majority of studies in order to allow the application of meaningful corrections. Conversely, the use of fever and any level of peripheral infection to define a malaria case corresponds closely to the criteria recommended for case treatment across the world [82],[83] and thus has congruence with disease burdens that should be managed with appropriate medicines. Finally, we have not considered the impact of scaled or partial coverage of interventions aimed at preventing infection, because we feel this is reflected in the parasite prevalence surface [41]. The one exception is the use of failing monotherapy because recrudescent cases will not be reflected in our endemicity–incidence relationship based on active case detection with effective treatment and thus, where this poses a significant threat, our estimates will be even greater underestimates. Despite the caveats, we believe that this approach to *P. falciparum* disease burden estimation provides an alternative and, in nations with inadequate surveillance, the only existing approach to estimating the true global risk of malaria.

### Robust Estimates of Uncertainty

We have used joint simulations from an established Bayesian geostatistical model for *P. falciparum* parasite prevalence in the 2- up to 10-year age group (*Pf*PR_2–10_) (Figure 2), integrated with a second Bayesian model for the endemicity-incidence relationship (Figures 6 and 7), to generate spatially distributed estimates of the clinical burden of *P. falciparum* malaria worldwide with associated uncertainty. This reflects the uncertainty in measures of risk that results in a range of possible estimates globally from 349 to 553 million cases in 2007; similar to the range size in other malaria burden estimations [1],[3],[5],[7],[84]. This elaborate modelling framework has allowed the incorporation of uncertainty in our knowledge of the intensity of transmission at any given location with uncertainty in our knowledge of how this intensity influences the rate of clinical episodes at that location, allowing the net uncertainty to be propagated into final estimates of clinical burden. Crucially, the joint simulation framework allows modelled uncertainty to be aggregated across regions to provide our final credible intervals for country and region-specific burden estimates, a procedure that is not possible using the per-pixel prediction approaches currently pervasive in disease mapping.

The WHO has recently used surveillance-based techniques to estimate the combined burden of *P. falciparum* and *P. vivax* to be 247 million cases in 2006 (189–287) [8]. The WHO placed greater reliance on data reported routinely through national health management information systems (HMIS), which were subjected to a range of evidence-based adjustments for nonattendance, reporting rates, and diagnostic practices. These HMIS data were used for national estimates in 77 of 107 countries considered worldwide (Protocol S2). The fidelity of these estimates and their sensitivity to assumptions underlying the suite of adjustment factors was dependent on the quality and completeness of the HMIS data from each country. In the 30 countries with the least reliable national data, a predecessor of the prevalence-based modelling protocol presented in this study was used [8],[85]. The results are shown for individual countries in Protocol S2. These estimates were revised in 2009 but data have not been made available for all countries [9].

### Uncertainty in India

India is a country of considerable diversity in its current and historic malaria ecology, a country which suffered in excess of a million deaths PA during the colonial era [86]. Since its independence in 1947, India has achieved remarkable malaria control gains, reducing morbidity to 100,000 cases and mortality to zero in 1965 [87] at the peak of the Global Malaria Eradication Programme [53]. Since this time malaria resurgence has been widely reported in the country [87]–[89]. The contemporary burden is unknown [90]–[97] and is probably exacerbated by the unique problem of urban malaria, maintained by *Anopheles stephensi* [49],[88],[98].

India remains a massive source of uncertainty in our cartography-based estimates (Results and Protocol S2), contributing over three-quarters (76%) of the uncertainty range in the global incidence estimates. It is therefore important to explore ancillary evidence for the plausibility of these cartographic estimates of 102 (31–187) million compared to the much smaller estimate derived from surveillance-based techniques: 10.65 (9.00–12.41) million [8].

A wide range of factors can reduce the accuracy of surveillance data. Low rates of care-seeking for malaria in the formal health sector, unreliable diagnoses, poor record keeping, and inefficient data transfer and collation systems can all combine to make the number of cases formally reported a small fraction of the true number of cases in a population. To mitigate these substantial sources of bias in raw surveillance data, the approach taken by WHO is to modify the raw data using a number of adjustment parameters, which can include the proportion of people with fever seeking formal-sector care, the reporting rate by facilities, and the likely positivity rates amongst non-attending and non-slide–confirmed cases of fever [8],[85]. Such adjustments are essential, but the validity of the final estimate is entirely dependent on the values used for each parameter, which are drawn from a mixture of health-system reported figures, secondary data of varying fidelity, and ad-hoc decision rules. A key weakness of this approach is that, in many cases, the true uncertainty around key parameter values is not captured adequately.

In the case of India, raw surveillance data for 2006 reported 1.8 million malaria cases. Adjustments were made for care-seeking behaviour and reporting rate by health facilities, which combined to increase the estimate by a factor of 5.0–6.9, to the final figure of 10.65 (9.00–12.41) million [8], with the confidence range primarily reflecting differing assumptions for positivity rate amongst nonpresenting fevers. Assessing the validity of either the individual adjustment parameters or the final estimate is difficult since, by definition, gold-standard values for comparison do not exist. However, numerous studies in India have compared case numbers detected via routine surveillance with parallel community-based longitudinal surveys and found disparities much larger than the factor of approximately six used by the WHO. For example, malaria incidence in the Kichha Primary Health Centre (PHC) and Kharkhoda PHC were 23.5 and 38.9 times under-reported, respectively [61]. Large discrepancies were also reported in Gadarpur PHC (53.5×) [62], Nichlaul PHC (20.3×) [64] and Ahmedabad City (9×) [65]. For India, the WHO estimate makes no allowance for misdiagnosis within the formal health sector, although studies have shown that this can be substantial. In the PHCs of ten districts in Uttar Pradesh, 75% of slide-confirmed infections were missed when the slides were checked by a reference centre [28], and an estimated 58% were missed in Bisra PHC when fortnightly rather than weekly surveillance was used [63].

In completely independent work, the final estimate for malaria mortality in India in 2006, taken from the “million deaths” verbal autopsy study was approximately 200,000 deaths (Dhingra N, et al., unpublished data). Assuming a conservative case fatality rate of only one per 1,000 [99],[100], this would lead to a morbidity estimate much closer to those retrieved using cartographic techniques—somewhere in the region of 200 million cases. Similar arguments of plausible morbidity totals can be made using other recent mortality estimates of 50,000 deaths in 1998 in 15 of 38 States and Union Territories [90],[93]. In sum, we find that cartography-based estimates are supported by, and resonate most closely with, the findings in the recent literature [90]–[96], although it should be acknowledged that there is likely to be a publication bias in reports of problems over progress.

There is no perfect post-hoc correction to compensate for poor malaria surveillance. Both methods using routine HMIS adjusted for nonattendance, poor reporting, and inadequate diagnostics, and those presented here, have limitations with respect to coverage and quality of the input data for each model, and with respect to underlying modelling assumptions. Both approaches to burden estimation result in wide margins of confidence and the inevitable plea from any such analysis is for accurate national reporting systems or more empirical epidemiological data. It can be seen clearly from these analyses that improvements in basic malariometric information in only four countries would radically reduce uncertainty in the global estimates of the malaria burden. Additionally, the approach presented does provide a standardized method across all malaria-endemic countries, using a set of transparent epidemiological rules allowing countries to be compared without concerns about differences in national health information quality or coverage.

### A Hybrid Approach?

To allay some of the concerns about the use of cartographic techniques in low-endemicity settings [101], we have also investigated the possibility of combining the two burden estimation processes for the 87 *Pf*MECs.

Seven countries have “gold-standard” reporting systems requiring no adjustment by either technique. These are in the African Regional Office (AFRO): South Africa; in AMRO: Belize and Panama; EMRO: Iran and Saudi Arabia; and EURO: Kyrgyzstan and Tajikistan (7/87). In many *Pf*MECs in the Africa+ region, an outdated cartographic technique was used by WHO [8]. Since the new methods outlined here are an unambiguous improvement, these were adopted for the following *Pf*MECs: in AFRO: Angola, Burkina Faso, Cameroon, Central African Republic, Chad, Congo, Côte d'Ivoire, DRC, Equatorial Guinea, The Gambia, Ghana, Guinea, Guinea-Bissau, Liberia, Malawi, Mali, Mauritania, Mozambique, Niger, Nigeria, Sierra Leone, Togo, Uganda, and Zimbabwe; and in EMRO: Yemen (25/87). In addition, Mayotte in AFRO and French Guiana in AMRO have no WHO estimates, so we default to the cartographic approach (2/87). Conversely there are two small island nations in AFRO (Cape Verde and the Comoros) for which we had no contemporary *Pf*PR data and the spatial resolution of mapping was not ideal, so the WHO estimates were used (2/87).

We then calculated, for all countries, the ratio of the width of the 95% credible interval to the point estimate obtained using the cartographic method and ranked this relative uncertainty metric by nation (Protocol S2). For those countries where this cartography-based uncertainty ranked in the bottom half (i.e., the least uncertain, corresponding to a ratio of <40), we adopted our cartographic-based estimates. They were in AFRO: Benin, Burundi, Ethiopia, Gabon, Kenya, Madagascar, Rwanda, Senegal, United Republic of Tanzania, and Zambia; in EMRO: Somalia and Sudan; in SEARO: India, Indonesia, and Myanmar; and in WPRO: Papua New Guinea (16/87). Conversely, in countries where cartography-based uncertainty was ranked in the top half (ratio ≥40) we defaulted to the WHO estimate. They were in AFRO: Botswana, Eritrea, Namibia, São Tomé and Príncipe, and Swaziland; in AMRO: Bolivia, Brazil, Colombia, Dominican Republic, Ecuador, Guatemala, Guyana, Haiti, Honduras, Nicaragua, Peru, Suriname, and Venezuela; in EMRO: Afghanistan, Djibouti, and Pakistan; in SEARO: Bangladesh, Bhutan, Nepal, Sri Lanka, Thailand, and Timor-Leste; and in WPRO: Cambodia, China, Lao People's Democratic Republic, Malaysia, Philippines, Solomon islands, Vanuatu, and Viet Nam (35/87).

This hybrid approach resulted in seven countries using gold standard national reports, 43 nations using cartographic techniques and 37 using the surveillance-based methods of WHO. The percentage of the global burden estimated by each technique was 0.001%, 97.722%, and 2.277%, respectively. Using a hybrid approach therefore makes very little difference to the global clinical burden estimate for 2007, although it has a significant impact on the absolute number of cases estimated for each country (Protocol S2).

### Interpreting Estimates

These estimates improve upon previous efforts, which used epidemiological approaches to estimate the global burden of *P. falciparum* clinical attacks in 2002 (515 million, interquartile range 300–660 million) [5], and more recent efforts to estimate paediatric clinical events due to high parasite densities of *P. falciparum* in Africa in 2000 (116 million, uncertainty interval 91–258 million) [7]. The differences between these results and previous efforts are not primarily due to differences in the base year of analysis or definitions of a clinical attack, but stem largely from differences in estimation of the endemicity-structured PARs. In our previous global estimates [5], we adapted a historical, categorical description of malaria endemicity, whilst in Africa we [1],[3],[4] and others [6],[7] have previously used a climate suitability model of the likelihood of stable transmission as an index of differences in transmission intensity [102],[103]. The single largest difference between previous work and the present iteration of *P. falciparum* disease burden estimation is that neither previous approach was based upon an empirically defined risk map of malaria transmission [41]. Comparing estimates derived using these different techniques, over various time periods, is not a sound basis for investigating trends and should be avoided.

It is clear that investing in radically improved surveillance and/or nationally representative malariometric surveys would substantially increase the fidelity of national and, by extension, global burden estimates. Because there are regional differences in the uncertain relationship between transmission intensity and disease outcome [42], more information derived from active case detection studies would improve the precision in our estimates of disease incidence within these transmission ranges. This information, while welcome, is likely to make only small differences to the computed risk in most scenarios of malaria transmission defined here. As a consequence, we believe that until there is a universally reliable reporting system for malaria cases worldwide to support comprehensive surveillance-based estimates, a concerted effort to map the changing spatial extents and intensity of transmission will remain a valuable contribution to the future estimations of a changing disease burden worldwide. In the short term, measuring how the “denominator” changes with time is clearly easier and cheaper than improving the global state of health information systems.

### Future Directions

Many improvements will be possible with further work. We have not stratified incidence by age nor considered any of the consequential morbid events, sequelae, or mortality. Systematic biases in the identification of the extent of stable and unstable transmission would clearly impact estimates, and developing the datasets and techniques to address this problem is an important avenue for future work. Nor have we modelled uncertainty in HMIS reporting in unstable and low-stable transmission zones, and this might be possible with a methodological hybrid combining higher spatial resolution HMIS facility data with geostatistical techniques [37]. Moreover, we have not been able to consider some sources of uncertainty in the current framework; for example, those concerning the enumeration of the underlying population, based on collated census data; urban extent maps; and UN population projections. Finally, we have not considered the morbid burden posed by *P. vivax*. There are important differences in the biology of *P. vivax* [104] which make its control [105], and thus cartography-based burden estimation, problematic: its tendency to cause relapses [106], the routine reliability of parasite diagnosis when coincidentally prevalent with *P. falciparum* [107],[108] and the less well-defined relationship between transmission intensity and disease outcome. These all make an informed cartography of *P. vivax* distribution and estimations of disease burden considerably more complex than for *P. falciparum*. We do not underestimate the likely disease burden of *P. vivax* malaria [109]–[112], but new, innovative approaches based on an understanding of the clinical epidemiology and better cartography are required to improve upon current efforts to define the burden due to *P. vivax*.

It is worth reiterating that if the international community wishes to demonstrate progress in malaria control, then the quantity and timeliness of prevalence information and parasite-specific surveillance records must dramatically improve. This is true for all countries but is particularly important in India, Nigeria, DRC, and Myanmar because of the large populations at risk and the paucity of existing malariometric information. These improvements in information collection and provision are as important across space (to be geographically representative of all transmission settings and intervention scenarios) as they are through time, so that impact can be evaluated in a timely manner. Conceptually, we also envisage that significant progress will be made in improving the accuracy of these estimates by hybridising cartographic and surveillance-based approaches. This would be best achieved by combining geopositioned HMIS facility data with geostatistical model outputs [37], so that the relative uncertainty of each can be compared and complementary information from both sources combined in a single coherent spatial framework. Globally, this is likely to be of particular utility in those areas of low and unstable transmission where surveillance capabilities are often more robust and correspondingly where prevalence data are often rare as the number of people needed to be sampled to find infections is prohibitive [12].

The malaria clinical burden estimates presented in this paper are driven by the underlying model of global prevalence [41]. This global malaria map is, to our knowledge, the first evidence-based attempt to define populations at risk of different levels of parasite transmission. It is needed in order to define the ranges of disease outcomes at a global scale and can serve as the benchmark for malaria disease burden estimations. The map will inevitably change with time as new information on the spatial extents of transmission and new *Pf*PR_2–10_ data become increasingly available with the scale-up of interventions. The time–space functionality of the geostatistical model will increasingly capture the effects of scaled intervention efforts to reduce transmission, causing the size of the *Pf*PR used to compute disease burden to change. Revising the limits and endemicity maps from this baseline and propagating these changes through to revised enumerations of clinical burden thus represents a useful complementary technique to assessing the impact of financing [113] on our progress towards international development targets for reducing malaria burden [59],[114].

## Supporting Information

Protocol S1 Supplemental methods.
(1.39 MB DOC)

Protocol S2 A comparison of cartographic and surveillance-based estimates of national clinical incidence.
(0.34 MB DOC)

## Abbreviations

Africa+: Africa, Yemen, and Saudi Arabia
AFRO: African Regional Office
AMRO: American Regional Office
CSE Asia: Central and South East Asia
DRC: Democratic Republic of the Congo
EMRO: Eastern Mediterranean Regional Office
EURO: European Regional Office
GRUMP: Global Rural-Urban Mapping Project
HMIS: health management information systems
MAP: Malaria Atlas Project
PA: per annum
PAR: population at risk
PHC: primary health centre
*Pf*API: *P. falciparum* annual parasite incidence
*Pf*MEC: *P. falciparum* malaria endemic country
*Pf*PR: *P. falciparum* parasite rate
SEARO: South East Asian Regional Office
WHO: World Health Organization
WPRO: Western Pacific Regional Office

## References

[1] Snow RW, Craig M, Deichmann U, Marsh K (1999). Estimating mortality, morbidity and disability due to malaria among Africa's non-pregnant population.. Bull World Health Organ.

[2] Carter R, Mendis KN (2002). Evolutionary and historical aspects of the burden of malaria.. Clin Microbiol Rev.

[3] Snow RW, Craig MH, Newton CRJC, Steketee RW (2003). The public health burden of *Plasmodium falciparum* malaria in Africa: deriving the numbers..

[4] Hay SI, Guerra CA, Tatem AJ, Atkinson PM, Snow RW (2005). Urbanization, malaria transmission and disease burden in Africa.. Nat Rev Microbiol.

[5] Snow RW, Guerra CA, Noor AM, Myint HY, Hay SI (2005). The global distribution of clinical episodes of *Plasmodium falciparum* malaria.. Nature.

[6] Rowe AK, Rowe SY, Snow RW, Korenromp EL, Schellenberg JRA (2006). The burden of malaria mortality among African children in the year 2000.. Int J Epidemiol.

[7] Roca-Feltrer A, Carneiro I, Armstrong Schellenberg JR (2008). Estimates of the burden of malaria morbidity in Africa in children under the age of 5 years.. Trop Med Int Health.

[8] WHO (2008). World malaria report 2008..

[9] WHO (2009). World malaria report 2009..

[10] Murray CJ, Lopez AD (1996). Global burden of disease and injury series..

[11] Murray CJL, Lopez AD (1997). Mortality by cause for eight regions of the world: Global Burden of Disease Study.. Lancet.

[12] Hay SI, Smith DL, Snow RW (2008). Measuring malaria endemicity from intense to interrupted transmission.. Lancet Infect Dis.

[13] Marsh K, Snow RW (1999). Malaria transmission and morbidity.. Parassitologia.

[14] Trape JF, Rogier C (1996). Combating malaria morbidity and mortality by reducing transmission.. Parasitol Today.

[15] Snow RW, Marsh K (1998). New insights into the epidemiology of malaria relevant for disease control.. Br Med Bull.

[16] Smith TA, Leuenberger R, Lengeler C (2001). Child mortality and malaria transmission intensity in Africa.. Trends Parasitol.

[17] Snow RW, Marsh K (2002). The consequences of reducing transmission of *Plasmodium falciparum* in Africa.. Adv Parasitol.

[18] Smith T, Killeen G, Lengeler C, Tanner M (2004). Relationships between the outcome of *Plasmodium falciparum* infection and the intensity of transmission in Africa.. Am J Trop Med Hyg.

[19] Genton B, Smith T, Baea K, Narara A, al-Yaman F (1994). Malaria: how useful are clinical criteria for improving the diagnosis in a highly endemic area?. Trans R Soc Trop Med Hyg.

[20] Smith T, Hurt N, Teuscher T, Tanner M (1995). Is fever a good sign for clinical malaria in surveys of endemic communities?. Am J Trop Med Hyg.

[21] Chandramohan D, Jaffar S, Greenwood B (2002). Use of clinical algorithms for diagnosing malaria.. Trop Med Int Health.

[22] da Silva-Nunes M, MR S, LB A, Souza EA, Martins LC (2006). The Acre Project: the epidemiology of malaria and arthropod-borne virus infections in a rural Amazonian population.. Cad Saude Publica.

[23] Koram KA, Molyneux ME (2007). When is “malaria” malaria? The different burdens of malaria infection, malaria disease, and malaria-like illnesses.. Am J Trop Med Hyg.

[24] Amexo M, Tolhurst R, Barnish G, Bates I (2004). Malaria misdiagnosis: effects on the poor and vulnerable.. Lancet.

[25] Reyburn H, Mbatia R, Drakeley C, Carneiro I, Mwakasungula E (2004). Overdiagnosis of malaria in patients with severe febrile illness in Tanzania: a prospective study.. Br Med J.

[26] Smith T, Schellenberg JA, Hayes R (1994). Attributable fraction estimates and case definitions for malaria in endemic areas.. Stat Med.

[27] Chaturvedi HK, Mahanta J, Pandey A (2009). Treatment-seeking for febrile illness in north-east India: an epidemiological study in the malaria endemic zone.. Malar J.

[28] Choudhury DS, Sharma VP, Bhalla SC, Aggarwal SS, Das SK (1987). Malaria prevalence in patients attending primary health centres in ten districts of Uttar Pradesh.. Indian J Malariol.

[29] Abeysekera T, Wickremasinghe AR, Gunawardena DM, Mendis KN (1997). Optimizing the malaria data recording system through a study of case detection and treatment in Sri Lanka.. Trop Med Int Health.

[30] Donnelly MJ, Konradsen F, Birley MH (1997). Malaria-treatment-seeking behaviour in the southern Punjab, Pakistan.. Ann Trop Med Parasitol.

[31] Al-Laham H, Khoury R, Bashour H (2001). Reasons for underreporting of notifiable diseases by Syrian paediatricians.. East Mediterr Health J.

[32] Chilundo B, Sundby J, Aanestad M (2004). Analysing the quality of routine malaria data in Mozambique.. Malar J.

[33] Murray CJ, Lopez AD, Wibulpolprasert S (2004). Monitoring global health: time for new solutions.. Br Med J.

[34] Branch O, Casapia WM, Gamboa DV, Hernandez JN, Alava FF (2005). Clustered local transmission and asymptomatic *Plasmodium falciparum* and *Plasmodium vivax* malaria infections in a recently emerged, hypoendemic Peruvian Amazon community.. Malar J.

[35] Oum S, Chandramohan D, Cairncross S (2005). Community-based surveillance: a pilot study from rural Cambodia.. Trop Med Int Health.

[36] Stansfield S (2005). Structuring information and incentives to improve health.. Bull World Health Organ.

[37] Gething PW, Noor AM, Gikandi PW, Ogara EAA, Hay SI (2006). Improving imperfect data from health management information systems in Africa using space-time geostatistics.. PLoS Med.

[38] Kunimitsu A (2009). The accuracy of clinical malaria case reporting at primary health care facilities in Honiara, Solomon Islands.. Malar J.

[39] Lysenko AJ, Semashko IN, Lebedew AW (1968). Geography of malaria. A medico-geographic profile of an ancient disease [in Russian].. Itogi Nauki: Medicinskaja Geografija.

[40] Guerra CA, Gikandi PW, Tatem AJ, Noor AM, Smith DL (2008). The limits and intensity of *Plasmodium falciparum* transmission: implications for malaria control and elimination worldwide.. PLoS Med.

[41] Hay SI, Guerra CA, Gething PW, Patil AP, Tatem AJ (2009). A world malaria map: *Plasmodium falciparum* endemicity in 2007.. PLoS Med.

[42] Patil AP, Okiro EA, Gething PW, Guerra CA, Sharma SK (2009). Defining the relationship between *Plasmodium falciparum* parasite rate and clinical disease: statistical models for disease burden estimation.. Malar J.

[43] Gething PW, Patil AP, Hay SI (2010). Quantifying aggregated uncertainty in *Plasmodium falciparum* malaria prevalence and populations at risk *via* efficient space-time geostatistical joint simulation.. PLoS Comput Biol.

[44] Balk DL, Deichmann U, Yetman G, Pozzi F, Hay SI (2006). Determining global population distribution: methods, applications and data.. Adv Parasitol.

[45] U.N.P.D (2006). http://esa.un.org/unpp/.

[46] Hay SI, Noor AM, Nelson A, Tatem AJ (2005). The accuracy of human population maps for public health application.. Trop Med Int Health.

[47] Macdonald G (1957). Local features of malaria..

[48] Mouchet J, Carnevale P, Coosemans M, Julvez J, Manguin S (2004). Paludisme et grandes régions biogéographiques..

[49] Hay SI, Sinka ME, Okara RM, Kabaria CK, Mbithi PM (2010). Developing global maps of the dominant *Anopheles* vectors of human malaria.. PLoS Med.

[50] Pull JH (1972). Malaria surveillance methods, their uses and limitations.. Am J Trop Med Hyg.

[51] Ray AP, Beljaev AE (1984). Epidemiological surveillance: a tool for assessment of malaria and its control.. J Commun Dis.

[52] Molineaux L, Muir DA, Spencer HC, Wernsdorfer WH, Wernsdorfer WH, McGregor I (1988). The epidemiology of malaria and its measurement.. Malaria: principles and practice of malariology.

[53] Pampana E (1969). A textbook of malaria eradication..

[54] Swaroop S, Gilroy AB, Uemura K (1966). Statistical methods in malaria eradication..

[55] Yekutiel P (1960). Problems of epidemiology in malaria eradication.. Bull World Health Organ.

[56] WHO (1963). Terminology of malaria and of malaria eradication.. Report of a drafting committee.

[57] Macauley C (2005). Aggressive active case detection: a malaria control strategy based on the Brazilian model.. Soc Sci Med.

[58] WHO (2007). Malaria elimination: a field manual for low and moderate endemic countries..

[59] R.B.M.P (2008). The global malaria action plan for a malaria free world..

[60] Feachem RGA, Phillips AA, Targett GA, on behalf of the Malaria Elimination Group (2009). Shrinking the Malaria Map: a Prospectus on Malaria Elimination..

[61] Sharma VP, Choudhury DS, Ansari MA, Malhotra MS, Menon PKB (1983). Studies on the true incidence of malaria in Kharkhoda (District Sonepat, Haryana) and Kichha (District Nainital, U.P.) Primary Health Centres.. Indian J Malariol.

[62] Malhotra MS, Shukla RP, Sharma VP (1985). Studies on the incidence of malaria in Gadarpur town of Terai, distt.Nainital U.P.. Indian J Malariol.

[63] Ghosh SK, Kumar A, Chand SK, Choudhury DS (1989). A preliminary malaria survey in Bisra PHC, district Sundergarh, Orissa.. Indian J Malariol.

[64] Joshi PL, Chandra R, Bhattacharya M (1999). An outbreak of malaria in district Maharajganj: an outcome of neglected surveillance.. J Commun Dis.

[65] Yadav RS, Bhatt RM, Kohli VK, Sharma VP (2003). The burden of malaria in Ahmedabad city, India: a retrospective analysis of reported cases and deaths.. Ann Trop Med Parasitol.

[66] Erhart A, Thang ND, Xa NX, Thieu NQ, Hung LX (2007). Accuracy of the health information system on malaria surveillance in Vietnam.. Trans R Soc Trop Med Hyg.

[67] Incardona S, Vong S, Chiv L, Lim P, Nhem S (2007). Large-scale malaria survey in Cambodia: novel insights on species distribution and risk factors.. Malar J.

[68] Kidson C, Indaratna K (1998). Ecology, economics and political will: the vicissitudes of malaria strategies in Asia.. Parassitologia.

[69] Mouchet J, Carnevale P, Coosemans M, Julvez J, Manguin S (2004). Biodiversité du paludisme dans le monde..

[70] Talisuna AO, Bloland P, D'Alessandro U (2004). History, dynamics, and public health importance of malaria parasite resistance.. Clin Microbiol Rev.

[71] Gunewardena DM, Carter R, Mendis KN (1994). Patterns of acquired anti-malarial immunity in Sri Lanka.. Mem Inst Oswaldo Cruz.

[72] Maitland K, Williams TN, Newbold CI (1997). *Plasmodium vivax* and *P. falciparum*: biological interactions and the possibility of cross-species immunity.. Parasitol Today.

[73] Weatherall D, Akinyanju O, Fucharoen S, Olivieri N, Musgrove P, Jamison DT, Breman JG, Measham AR, Alleyne G, Claeson M (2006). Inherited disorders of hemoglobin..

[74] WHO/PAHO (2006). Regional strategic plan for malaria in the Americas 2006-2010..

[75] WHO/Regional Office for Europe (2008). WHO meeting on progress achieved with malaria elimination in the WHO European Region..

[76] WHO/Regional Office for the Eastern Mediterranean (2007). Strategic plan for malaria control and elimination in the WHO Eastern Mediterranean Region 2006–2010..

[77] WHO/Regional Office for the Eastern Mediterranean (2007). Guidelines on the elimination of residual foci of malaria transmission..

[78] Brooker S, Clements ACA, Hotez PJ, Hay SI, Tatem AJ (2006). The co-distribution of *Plasmodium falciparum* and hookworm among African schoolchildren.. Malar J.

[79] Abu-Raddad LJ, Patnaik P, Kublin JG (2006). Dual infection with HIV and malaria fuels the spread of both diseases in sub-Saharan Africa.. Science.

[80] Pearce RJ, Pota H, Evehe MS, Ba el H, Mombo-Ngoma G (2009). Multiple origins and regional dispersal of resistant dhps in African Plasmodium falciparum malaria.. PLoS Med.

[81] Bejon P, Mwangi T, Lowe B, Peshu N, Hill AV (2007). Clearing asymptomatic parasitaemia increases the specificity of the definition of mild febrile malaria.. Vaccine.

[82] WHO (2006). Guidelines for the treatment of malaria..

[83] WHO (2010). Guidelines for the treatment of malaria. Second edition..

[84] Korenromp EL (2004). Malaria incidence estimates at country level for the year 2004-proposed estimates and draft report..

[85] Cibulskis RE, Bell D, Christophel EM, Hii J, Delacollette C (2007). Estimating trends in the burden of malaria at country level.. Am J Trop Med Hyg.

[86] Hehir P (1927). Malaria in India..

[87] Akhtar R, Learmonth A (1977). The resurgence of malaria in India 1965-76.. GeoJournal.

[88] Akhtar R, Dutt AK, Wadhwa V, Akhtar R, Dutt AK, Wadhwa V (2010). Malaria resurgence in urban India: lessons from health planning strategies.. Malaria in South Asia Eradication and Resurgence During the Second Half of the Twentieth Century.

[89] Sharma VP (1996). Re-emergence of malaria in India.. Indian J Med Res.

[90] Kumar A, Valecha N, Jain T, Dash AP (2007). Burden of malaria in India: retrospective and prospective view.. Am J Trop Med Hyg.

[91] Sharma VP (2007). Battling the malaria iceberg with chloroquine in India.. Malar J.

[92] Dash AP, Valecha N, Anvikar AR, Kumar A (2008). Malaria in India: challenges and opportunities.. J Biosci.

[93] Dash AP (2009). Estimation of true malaria burden in India..

[94] Diamond-Smith N, Singh N, Gupta RD, Dash A, Thimasarn K (2009). Estimating the burden of malaria in pregnancy: a case study from rural Madhya Pradesh, India.. Malar J.

[95] Sharma VP (2009). Hidden burden of malaria in Indian women.. Malar J.

[96] Singh N, Dash A, Thimasarn K (2009). Fighting malaria in Madhya Pradesh (Central India): Are we loosing the battle?. Malar J.

[97] Cohen AA, Dhingra N, Jotkar RM, Rodriguez PS, Sharma VP (2010). The Summary Index of Malaria Surveillance (SIMS): a stable index of malaria within India.. Popul Health Metr.

[98] Wadhwa V, Akhtar R, Dutt AK, Akhtar R, Dutt AK, Wadhwa V (2010). The dynamics of urban malaria in India: an update.. Malaria in South Asia Eradication and Resurgence During the Second Half of the Twentieth Century.

[99] Luxemburger C, Ricci F, Nosten F, Raimond D, Bathet S (1997). The epidemiology of severe malaria in an area of low transmission in Thailand.. Trans R Soc Trop Med Hyg.

[100] Sharma PK, Ramanchandran R, Hutin YJ, Sharma R, Gupte MD (2009). A malaria outbreak in Naxalbari, Darjeeling district, West Bengal, India, 2005: weaknesses in disease control, important risk factors.. Malar J.

[101] Snow RW, Guerra CA, Noor AM, Myint HY, Hay SI (2005). Malaria risk: Snow *et al*. reply.. Nature.

[102] Craig MH, Snow RW, le Sueur D (1999). A climate-based distribution model of malaria transmission in sub-Saharan Africa.. Parasitol Today.

[103] Small J, Goetz SJ, Hay SI (2003). Climatic suitability for malaria transmission in Africa, 1911-1995.. Proc Natl Acad Sci U S A.

[104] Coatney GR, Collins WE, Warren M, Contacos PG (2003). The primate malarias..

[105] Sattabongkot J, Tsuboi T, Zollner GE, Sirichaisinthop J, Cui L (2004). *Plasmodium vivax* transmission: chances for control?. Trends Parasitol.

[106] Garnham PCC, Wernsdorfer WH, McGregor I (1988). Malaria parasites of man: life-cycles and morphology (excluding ultrastructure).. Malaria: principles and practice of malariology.

[107] Mayxay M, Pukrittayakamee S, Newton PN, White NJ (2004). Mixed-species malaria infections in humans.. Trends Parasitol.

[108] Rosenberg R (2007). *Plasmodium vivax* in Africa: hidden in plain sight?. Trends Parasitol.

[109] Mendis K, Sina BJ, Marchesini P, Carter R (2001). The neglected burden of *Plasmodium vivax* malaria.. Am J Trop Med Hyg.

[110] Baird JK (2007). Neglect of *Plasmodium vivax* malaria.. Trends Parasitol.

[111] Price RN, Tjitra E, Guerra CA, Yeung S, White NJ (2007). Vivax malaria: neglected and not benign.. Am J Trop Med Hyg.

[112] Mueller I, Galinski MR, Baird JK, Carlton JM, Kochar DK (2009). Key gaps in the knowledge of Plasmodium vivax, a neglected human malaria parasite.. Lancet Infect Dis.

[113] Snow RW, Guerra CA, Mutheu JJ, Hay SI (2008). International funding for malaria control in relation to populations at risk of stable *Plasmodium falciparum* transmission.. PLoS Med.

[114] Attaran A (2005). An immeasurable crisis? A criticism of the millennium development goals and why they cannot be measured.. PLoS Med.

