# Supplementary material for: Estimating the Global Clinical Burden of Plasmodium falciparum Malaria in 2007
Source: PLoS Med. 2010 Jun 15;7(6):e1000290. doi: 10.1371/journal.pmed.1000290 (PMC2885984; doi:10.1371/journal.pmed.1000290)
Supplement: Protocol S1 — Supplemental methods. (1.39 MB DOC) [file pmed.1000290.s001.doc]

### **Protocol S1: Supplemental Methods**

### **S1.1. Statistical Model for *P. falciparum* Prevalence**

Full details of the geostatistical model for *P. falciparum* prevalence are provided in the supplementary information: “Protocol S3” of Hay *et al*. (2009) [1]. Briefly, the th observation was modelled as binomially distributed, conditional on the sample size , the age limits and a spatiotemporal random field evaluated at the location and time of the survey. The “typical” prevalence was a composition of a link function, in this case the standard inverse-logit, and a Gaussian process denoted . The random function mapped and to community prevalence within the age limits of survey . The distributional parameters of the unknown function were a mean function and a covariance function , where denotes scalar parameters, including covariate coefficients [2,3]. The model was completed by priors for . In probability notation:

(SA.1)

For clarity, this section will refer to this schematic representation of the model.

A major reason for the success of Gaussian processes in geostatistical modelling is the fact that they are trivial to marginalize. The vector obtained by evaluating at all the survey locations and times has a multivariate normal distribution:

(SA.2)

The vector is constructed in the same way as , and the matrix is defined as:

(SA.3)

Conditional on , is independent of evaluated at all other locations, so this marginalization can be used to reduce to a simple multivariate normal variable at the model-fitting stage.

##### Sampling the Posterior Predictive Distribution

The model was fitted using the Markov chain Monte Carlo (MCMC) algorithm [4], which produces a sequence of samples from the joint posterior of and . Conditional on one of these samples, the value of at unobserved location and time can be sampled from its posterior predictive distribution:

(SA.4)

where the posterior predictive parameters are given by the standard conditioning formulas for multivariate normal variables [5]:

(SA.5)

These formulas apply regardless of whether denotes multiple prediction locations or a single point. Samples from the predictive distribution for many values of and from the MCMC trace can be regarded as samples from the target predictive distribution [4].

The global *P. falciparum* endemicity maps [1] were summaries of the posterior predictive distribution of an annual average at year and pixels :

(SA.6)

where denotes the age range , which has been found previously to be highly responsive to transmission intensity [6]. These annual averages were preferred to the more standard point evaluation:

(SA.7)

because malaria transmission is known to be seasonal, meaning no particular point in time adequately captures the overall annual transmission pattern.

### Statistical model relating *P. falciparum* prevalence and clinical incidence

The empirical prevalence-incidence model presented recently [7] modelled the relationship between *P. falciparum* prevalence and observed clinical incidence as a negative binomial parameterised by the average parasite rate over the survey period in the 2-10 year old age group, and the effective length of the survey, :

(SA.8)

The function was an increasing parabolic function of average parasite rate, and the mean incidence was assigned a Gaussian process prior. The posterior distribution of the random function , which gave the overall relationship between average parasite rate and incidence, was used directly in the current work. This posterior distribution is illustrated in Figure A2.

### **S1.2. Volumetric Prediction and Joint Simulation**

To produce maps summarizing the predictive distribution of a point evaluation, such as Eq. (SA.4), at a single point in time, it suffices to follow the procedure outlined above independently for each pixel. That is, the predictive distributions , , at the pixels in the output raster grid can be sampled and summarized independently. The computational cost of producing such a map is proportional to the number of pixels in the raster, for large raster grids.

Producing maps summarizing temporal integrals like Eq. (SA.6) is more involved. Due to the nonlinear link function, the integral must be approximated as a discrete sum [8] of evaluations at a vector of time points spaced evenly between and . The pixels can still be considered independently, but it is not sufficient to treat the time points within independently in a given pixel. This is easy to understand by considering a limiting case: if is very fine, the discrete sum should produce a very good approximation to the integral. However, if is treated as independent at each point in , the sum amounts to taking the mean of a large number of independent random variables, whose individual standard deviations are inversely proportional to their number. The variance of this mean will be very small: in other words, for very fine the sum is nearly determined by and . This is clearly incorrect. Predictive samples for the discrete sum must be based on joint predictive samples of .

The computational cost of the original *P. falciparum* endemicity maps [1] was proportional to the number of pixels in the raster grid, but the constant of proportionality was larger than it would be for maps based on point evaluations. At every pixel, and for every sample of and from the MCMC trace, the covariance matrix had to be constructed and its Cholesky decomposition had to be computed [9]. The computational costs of these operations were proportional to the square and the cube of the length of , respectively. However, since the length of was much smaller than the size of the dataset , these *P. falciparum* endemicity maps [1] were not much more expensive to compute than maps based on point evaluations.

The burden estimates presented in this paper are also based on predictive distributions of integrals:

(A9)

where is the administrative unit or other geographical region in which total burden is to be computed, is the random function mapping prevalence to clinical incidence and is the GRUMP population surface [10] at year . Like Eq. (SA.6), this is a volumetric quantity that can be estimated as a discrete sum.

However, the scale is massively different. The current paper and planned future modelling work by the Malaria Atlas Project (MAP, http:www.map.ox.ac.uk), require aggregate burden estimates and population-weighted average prevalences for any desired spatial region, from individual pixels to continents, and over any time interval between 1985 (corresponding to the oldest points in the MAP database) and the present. A raster grid spanning the malarious regions of Africa at 5 × 5 km spatial resolution and monthly temporal resolution contains approximately 345 million pixels. An covariance evaluation (where denotes that the computational cost of this evaluation is proportional to the square of the number of prediction locations) is completely infeasible on this scale, let alone an Cholesky decomposition. Block circulant embedding [11] can be used to sample multivariate normal variables in operations, but could not be adapted to our situation: reflecting the autocovariance array along three axes requires more memory than was available and, more importantly, due to the curvature of the Earth, the covariance matrix cannot be put in block Toeplitz form [11].

To overcome this computational hurdle, an application-specific algorithm was designed, which is described in full elsewhere [12]. In brief, the algorithm builds up the evaluations by scanning over time and space, taking advantage of the empirical fact that most of the information relevant to a particular scan-line is contained in nearby scan-lines. Although it is much faster than standard methods, the algorithm is expensive enough that we were forced to distribute the computation over a cluster of computers. Using this algorithm, we produced 500 joint simulations over , where indicates a 5 × 5 km pixel resolution surface over the *P. falciparum* malarious regions of the world. The simulations and subsequent reductions to maps and/or predictive distributions each took roughly two days of computer time for each of the three global regions and were distributed over compute instances on the Amazon Elastic Compute Cloud (http://aws.amazon.com/ec2).

### **References**

1. Hay SI, Guerra CA, Gething PW, Patil AP, Tatem AJ, et al. (2009) A world malaria map: *Plasmodium falciparum* endemicity in 2007. PLoS Med 6: e1000048.

2. Abrahamsen P (1997) A review of Gaussian random fields and correlation functions. Blindern, Oslo, Noway: Norwegian Computing Centre. 64 p.

3. Williams CKI (1997) Prediction with Gaussian process: from linear regression to linear prediction and beyond. Technical Report NCRG/97/012. Birmingham, U.K.: Neural Computing Research Group, Department of Computer Science and Applied Mathematics, Aston University. 19 p.

4. Gilks WR, Spiegelhalter DJ (1999) Markov Chain Monte Carlo in practice. Interdisciplinary Statistics. Boca Raton, Florida, U.S.A.: Chapman & Hall / CRC Press LLC.

5. West M, Harrison J (1997) Bayesian forecasting and dynamic models. New York, U.S.A.: Springer-Verlag New York, Inc.

6. Smith DL, Guerra CA, Snow RW, Hay SI (2007) Standardizing estimates of the *Plasmodium falciparum* parasite rate. Malar J 6: 131.

7. Patil AP, Okiro EA, Gething PW, Guerra CA, Sharma SK, et al. (2009) Defining the relationship between *Plasmodium falciparum* parasite rate and clinical disease: statistical models for disease burden estimation. Malar J 8: 186.

8. Burden RL, Faires DJ (2004) Numerical analysis. Pacific Grove, California, U.S.A.: Brooks/Cole Publishing Company.

9. Golub GH, van Loan CF (1996) Matrix computations. Baltimore, Maryland, U.S.A.: Johns Hopkins University Press.

10. Balk DL, Deichmann U, Yetman G, Pozzi F, Hay SI, et al. (2006) Determining global population distribution: methods, applications and data. Adv Parasitol 62: 119-156.

11. Dietrich CR, Newsam GN (1997) Fast and exact simulation of stationary Gaussian processes through circulant embedding of the covariance matrix. SIAM Journal on Scientific Computing 18: 1088-1107.

12. Gething PW, Patil AP, Hay SI (2010) Quantifying aggregated uncertainty in *Plasmodium falciparum* malaria prevalence and populations at risk *via* efficient space-time geostatistical joint simulation. PLoS Comput Biol 6: e1000724.
