# Supplementary material for: Estimating the Global Clinical Burden of Plasmodium falciparum Malaria in 2007
Source: PLoS Med. 2010 Jun 15;7(6):e1000290. doi: 10.1371/journal.pmed.1000290 (PMC2885984; doi:10.1371/journal.pmed.1000290)
Supplement: Protocol S2 — A comparison of cartographic and surveillance-based estimates of national clinical incidence. (0.34 MB DOC) [file pmed.1000290.s002.doc]

### **Protocol S2. A Comparison of Cartographic and Surveillance based estimates of National Clinical Incidence.**

This section provides a detailed summary of the estimated clinical burden of *P. falciparum* malaria (Table S2.1), showing for each of the 87 countries defined as endemic for *P. falciparum* [1], the estimated total cases with their 95% credible intervals (CIs). Sub-totals listing estimated case numbers stratified by endemicity class are also provided. In addition, national estimates and CIs are also graphed for each country (Figure S2), alongside an equivalent estimate made by the World Health Organization (WHO), and published in the World Malaria Report 2008 [2]. These estimates were revised in 2009 but data have not been made available for all countries [3]. The WHO estimates were based on two methodologies [4]. Data reported routinely through national health management information systems (hereafter the WHO method) was used in approximately three-quarters of the countries. The numbers were adjusted for non-attendance, incomplete reporting and misdiagnosis. The remaining countries (all in Africa) used an empirical model-based estimate that applied plausible incidence rate ranges [5] to populations at risk defined by climate suitability for *P. falciparum* malaria transmission [6], as advised by the Child Health Epidemiology Reference Group (hereafter the CHERG method).

The total global burden for 2007 estimated in this study (451 million, CI = 349-553 million) differs substantially from that presented by the WHO using the combined methods outlined above, who estimated a joint global burden for *P. falciparum* and *P. vivax* of 247 million cases in 2006 (CI = 189-287) [2]. When the respective estimates for individual countries presented in Figure S2 and Table S2.1 are examined, however, it is clear that in many cases there is a close correspondence.

In the countries of the Americas, both approaches predict uniformly low burden, with the largest contributions from Brazil and Haiti. Our estimated total burden for the Americas exceeds that of the WHO by 0.4 million, or some 15%.

In the expanded Africa region, most estimates are broadly similar, with a few notable exceptions. The pattern of differences also differs depending on the choice of method used by the WHO. Our estimates in those countries for which the WHO used the CHERG method tended to be larger, such that our sub-total for these countries alone exceeded that of the WHO by 54.0 million, or 33%. The largest individual differences for these countries were in Nigeria and the Democratic Republic of Congo, where our estimates of 73.2 and 29.1 million cases, respectively, are both around 25% larger than the respective WHO estimates of 58.0 and 23.6 million cases. For those countries where the WHO estimates were based on the HMIS method, the pattern was less clear, with some of our estimates substantially exceeding the WHO's estimates (for example, in Madagascar, where our estimate of 6.7 million cases is over 10 times larger than the WHO estimate of 0.6 million), and others being substantially smaller (for example, in Kenya and Ethiopia, where our estimates of 4.9 and 5.8 million cases are both less than half the respective WHO estimates of 11.3 and 12.4 million cases). Because of the mixed nature of the differences, our sub-total for these HMIS countries differed from that of the WHO by just 1.3 million cases, a relative difference of 2%. Overall, our estimated total burden for the Africa region exceeds that of the WHO by 52.6 million, or 24%.

It is in the Central and South East Asia (CSE Asia) region that our estimates are most consistently above those of the WHO, with our estimated total burden of 177.0 million cases for the region being more than six times larger than the 23.9 million estimated by the WHO. The disparity for individual countries grows in magnitude with the estimated burden, such that our estimates of 101.5, 26.1 and 12.3 million cases for the three largest contributors, India, Myanmar, and Indonesia, respectively, are 8.5, 5.2 and 3.9 times larger than those of the WHO, constituting between them a difference of 121.8 million cases. Indeed, the magnitude of these differences is such that, of the overall difference of 205 million cases in our respective global estimates, 75% is accounted for by the CSE Asia region as a whole, 60% by just these three countries, and 45% by India alone. It is clear that reducing the uncertainty in these global estimates and reconciling the differences between techniques will only be possible when the data used to make the estimates in these three countries is radically improved.

To enhance the comparability of these burden estimates, the number of clinical cases and their appropriate confidence intervals are also summarized by WHO region (Table S2.2).

**
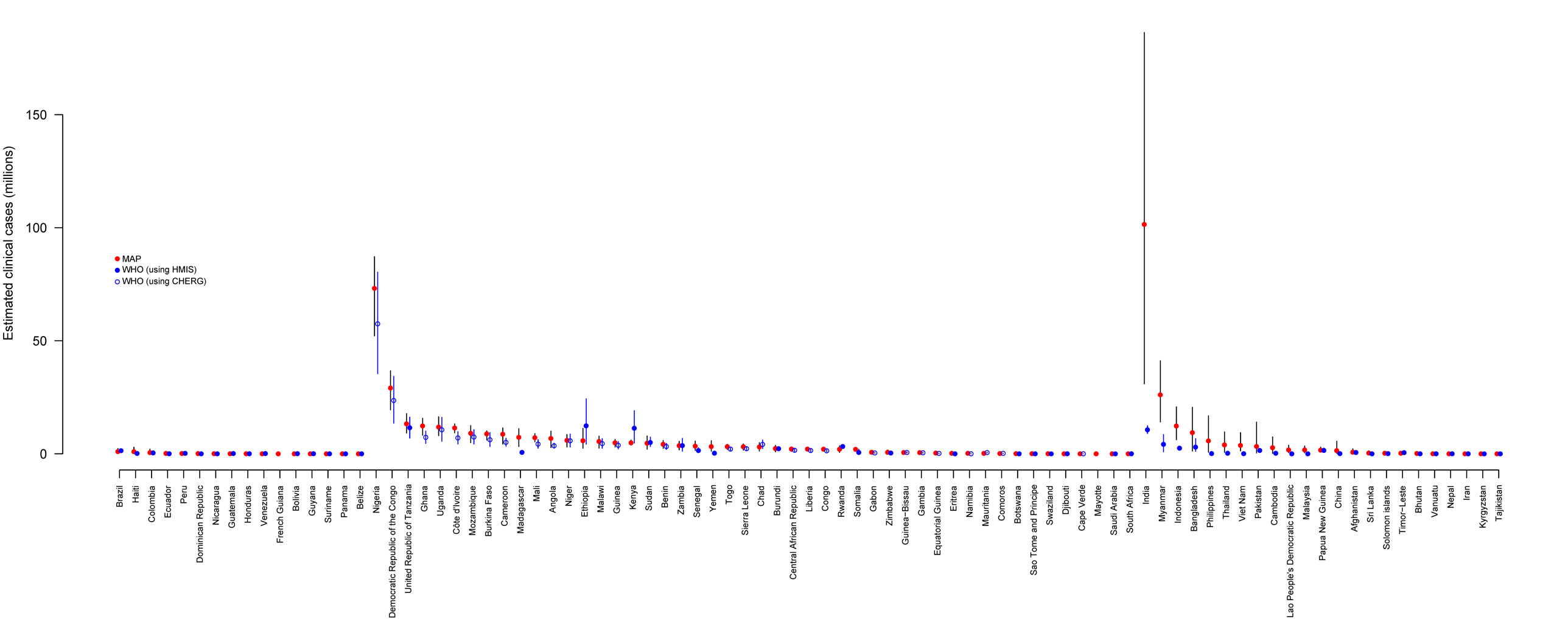
**

**Figure S2**. Estimated *P. falciparum* clinical cases by country in 2007. Red dots are the estimates (posterior means) made in this study by the Malaria Atlas Project (MAP). Black bars illustrate the associated 95% credible interval. Blue dots and rings are the World Health Organization (WHO) estimates from the World Malaria Report 2008 [2] using adjusted data from national health management information systems (HMIS) or the method adopted by the Child Health Epidemiology Reference Group (CHERG), respectively. Accompanying blue bars denote the range of the lower and upper estimates. Countries are grouped by region as defined in this study (left to right Americas, Africa+, CSE Asia) and then sorted in descending order of burden according to our estimates.

| **Table S2.1.** Estimated *P. falciparum* clinical cases (thousands) by country in 2007 | | | | | | |  |
| --- | --- | --- | --- | --- | --- | --- | --- |
|  | **Unstable risk** | ***Pf*PR_2-10_ ≤5%_2_** | ***Pf*PR_2-10_ >5-<40%_2_** | ***Pf*PR_2-10_ ≥40%_2_** | **Total** | **WHO_3_** | **CI ratio_4_** |
| **Africa + region_1_** |  |  |  |  |  |  |  |
| Nigeria | 0 (0-0) | 470 (6-1,788) | 16,108 (7,125-26,712) | 56,626 (25,313-78,780) | 73,203 (52,173-87,183) | 57,506 | 0.48 |
| Democratic Republic of the Congo | 0 (0-0) | 357 (29-880) | 7,379 (4,233-10,838) | 21,418 (8,337-32,257) | 29,155 (19,496-36,755) | 23,620 | 0.59 |
| United Republic of Tanzania | 0 (0-0) | 580 (283-1,004) | 5,843 (3,986-8,495) | 6,856 (3,171-12,170) | 13,279 (9,189-17,773) | 11,540 | 0.65 |
| Ghana | 0 (0-0) | 54 (0-279) | 2,783 (294-5,007) | 9,524 (3,991-15,076) | 12,360 (8,231-15,729) | 7,282 | 0.61 |
| Uganda | 0 (0-0) | 162 (3-530) | 5,526 (3,083-7,310) | 6,165 (2,217-13,716) | 11,853 (8,013-16,390) | 10,627 | 0.71 |
| Cote d'Ivoire | 0 (0-0) | 8 (0-70) | 1,092 (115-3,062) | 10,346 (6,403-12,793) | 11,447 (9,291-13,264) | 7,029 | 0.35 |
| Mozambique | 0 (0-0) | 207 (35-488) | 3,111 (1,275-4,512) | 5,751 (1,548-10,515) | 9,068 (4,930-12,460) | 7,433 | 0.83 |
| Burkina Faso | 0 (0-0) | 11 (0-88) | 1,004 (15-2,982) | 7,815 (3,328-9,931) | 8,830 (6,110-10,142) | 6,227 | 0.46 |
| Cameroon | 0 (0-0) | 77 (1-303) | 2,380 (908-4,095) | 6,236 (1,297-9,909) | 8,693 (4,347-11,438) | 5,091 | 0.82 |
| Madagascar | 0 (0-0) | 171 (0-411) | 2,536 (1,127-3,719) | 4,594 (886-9,757) | 7,302 (3,228-11,127) | 643 | 1.08 |
| Mali | 0.05 (0-0.47) | 59 (3-177) | 1,520 (456-2,666) | 5,575 (3,165-8,324) | 7,154 (5,326-8,960) | 4,317 | 0.51 |
| Angola | 0.03 (0-0.31) | 134 (2-358) | 1,937 (361-3,597) | 4,749 (615-9,490) | 6,820 (2,734-10,055) | 3,555 | 1.07 |
| Niger | 0.06 (0-0.62) | 107 (0-327) | 2,015 (885-3,043) | 3,826 (432-7,740) | 5,948 (3,019-8,537) | 5,760 | 0.93 |
| Ethiopia | 0.15 (0-1.50) | 1,252 (842-1,681) | 3,428 (1,144-6,262) | 1,103 (6-4,079) | 5,783 (2,482-11,252) | 12,405 | 1.52 |
| Malawi | 0 (0-0) | 133 (11-437) | 2,284 (989-3,725) | 3,060 (148-6,254) | 5,477 (2,519-7,844) | 4,528 | 0.97 |
| Guinea | 0 (0-0) | 36 (0-169) | 1,321 (293-2,434) | 3,529 (895-5,908) | 4,886 (2,999-6,393) | 3,766 | 0.69 |
| Kenya | 0.02 (0-0.17) | 504 (335-826) | 3,215 (2,508-3,998) | 1,144 (346-2,362) | 4,864 (3,872-6,109) | 11,342 | 0.46 |
| Sudan | 0.69 (0-6.87) | 706 (516-932) | 2,741 (1,050-4,538) | 1,249 (115-3,026) | 4,696 (2,037-7,885) | 5,023 | 1.24 |
| Benin | 0 (0-0) | 26 (0-186) | 967 (71-2,029) | 3,228 (827-5,597) | 4,221 (2,379-5,886) | 3,239 | 0.83 |
| Zambia | 0 (0-0) | 182 (58-361) | 1,846 (1,143-2,687) | 1,573 (256-3,414) | 3,601 (1,756-5,531) | 3,655 | 1.05 |
| Senegal | 0 (0-0) | 169 (11-448) | 1,962 (996-3,114) | 1,268 (134-3,435) | 3,398 (1,536-5,627) | 1,456 | 1.20 |
| Yemen | 0.55 (0-5.49) | 336 (107-620) | 2,234 (630-3,781) | 633 (0-2,244) | 3,203 (1,105-5,806) | 288 | 1.47 |
| Togo | 0 (0-0) | 11 (0-100) | 525 (0-1,263) | 2,636 (942-4,154) | 3,172 (1,951-4,223) | 2,086 | 0.72 |
| Sierra Leone | 0 (0-0) | 18 (0-166) | 696 (0-1,717) | 2,422 (388-4,202) | 3,135 (1,633-4,358) | 2,273 | 0.87 |
| Chad | 0.02 (0-0.16) | 153 (36-299) | 1,445 (723-2,237) | 1,435 (133-3,723) | 3,032 (1,240-4,937) | 4,179 | 1.22 |
| Burundi | 0 (0-0) | 42 (0-194) | 1,100 (359-1,757) | 1,207 (0-3,313) | 2,350 (909-3,712) | 2,271 | 1.19 |
| Central African Republic | 0 (0-0) | 25 (0-127) | 542 (83-1,081) | 1,526 (435-2,863) | 2,093 (1,106-3,031) | 1,574 | 0.92 |
| Liberia | 0 (0-0) | 5 (0-42) | 309 (0-872) | 1,767 (364-2,660) | 2,080 (1,113-2,716) | 1,460 | 0.77 |
| Congo | 0 (0-0) | 9 (0-68) | 345 (6-908) | 1,690 (448-2,743) | 2,044 (1,121-2,849) | 1,332 | 0.85 |
| Rwanda | 0 (0-0) | 46 (0-224) | 1,011 (266-1,727) | 969 (0-3,203) | 2,026 (607-3,535) | 3,251 | 1.45 |
| Somalia | 0.06 (0-0.55) | 245 (160-401) | 1,268 (774-1,807) | 477 (114-1,224) | 1,990 (1,272-2,800) | 609 | 0.77 |
| Gabon | 0 (0-0) | 6 (0-35) | 244 (20-508) | 426 (113-949) | 677 (346-1,085) | 387 | 1.09 |
| Zimbabwe | 0 (0-0) | 155 (35-235) | 353 (23-923) | 165 (0-553) | 674 (71-1,696) | 350 | 2.41 |
| Guinea-Bissau | 0 (0-0) | 16 (0-66) | 273 (40-574) | 280 (0-942) | 568 (102-1,036) | 603 | 1.64 |
| Gambia | 0 (0-0) | 22 (0-95) | 302 (65-619) | 211 (0-773) | 535 (144-1,010) | 469 | 1.62 |
| Equatorial Guinea | 0 (0-0) | 0 (0-0) | 50 (9-113) | 278 (196-379) | 328 (261-422) | 193 | 0.49 |
| Eritrea | 0.10 (0-0.96) | 90 (19-170) | 124 (2-617) | 11 (0-149) | 226 (24-840) | 19 | 3.62 |
| Namibia | 0.04 (0-0.39) | 27 (8-55) | 122 (4-295) | 70 (0-334) | 220 (15-496) | 35 | 2.19 |
| Mauritania | 0.04 (0-0.41) | 20 (7-35) | 108 (40-197) | 70 (2-242) | 198 (76-411) | 559 | 1.69 |
| Comoros | 0 (0-0) | 14 (0-47) | 66 (0-223) | 28 (0-283) | 109 (2-366) | 205 | 3.35 |
| Botswana | 0 (0-0) | 16 (4-33) | 44 (2-134) | 14 (0-108) | 75 (10-261) | 7 | 3.38 |
| São Tomé and Príncipe | 0 (0-0) | 1 (0-10) | 29 (0-81) | 31 (0-123) | 61 (9-135) | 10 | 2.08 |
| Swaziland | 0 (0-0) | 5 (0-14) | 27 (0-80) | 17 (0-133) | 49 (3-147) | 0 | 2.98 |
| Djibouti | 0.04 (0-0.45) | 0 (0-2) | 2 (0-8) | 0 (0-3) | 3 (0-12) | 39 | 3.99 |
| Mayotte | 0.03 (0-0.31) | 0 (0-0) | 0 (0-0) | 0 (0-0) | 0 (0-0) | 0 | - |
| Cape Verde | 0.03 (0-0.27) | 0 (0-0) | 0 (0-0) | 0 (0-0) | 0 (0-0) | 0 | - |
| Saudi Arabia^5^ | 0.467 | 0 (0-0) | 0 (0-0) | 0 (0-0) | 0 (0-0) | 1 | - |
| South Africa^5^ | 2.250 | 0 (0-0) | 0 (0-0) | 0 (0-0) | 0 (0-0) | 33 | - |
| **Table S2.1 cont.** Estimated *P. falciparum* clinical cases (thousands) by country in 2007 | | | | | | |  |
|  | **Unstable risk** | ***Pf*PR_2-10_ ≤5%_2_** | ***Pf*PR_2-10_ >5-<40%_2_** | ***Pf*PR_2-10_ ≥40%_2_** | **Total** | **WHO_3_** | **CI ratio_4_** |
| **Americas region_1_** |  |  |  |  |  |  |  |
| Brazil | 1.88 (0.00-18.75) | 414 (198-713) | 524 (22-1,874) | 36 (0-289) | 976 (265-2,392) | 1,379 | 2.16 |
| Haiti | 0.00 (0.00-0.00) | 462 (81-825) | 465 (0-2,242) | 22 (0-11) | 949 (81-2,939) | 165 | 3.01 |
| Colombia | 0.99 (0.00-9.92) | 181 (32-338) | 329 (0-1,838) | 22 (0-306) | 533 (32-2,063) | 408 | 3.80 |
| Ecuador | 0.17 (0.00-1.66) | 129 (10-306) | 46 (0-313) | 0 (0-0) | 175 (10-583) | 32 | 3.26 |
| Peru | 0.17 (0.00-1.73) | 91 (8-242) | 54 (0-382) | 0 (0-0) | 145 (10-547) | 222 | 3.70 |
| Dominican Republic | 0.29 (0.00-2.89) | 60 (9-115) | 42 (0-238) | 3 (0-0) | 105 (10-336) | 9 | 3.08 |
| Nicaragua | 0.21 (0.00-2.13) | 32 (2-114) | 16 (0-189) | 0 (0-0) | 49 (2-285) | 6 | 5.81 |
| Guatemala | 0.53 (0.00-5.34) | 19 (0-74) | 12 (0-68) | 13 (0-8) | 44 (1-141) | 141 | 3.10 |
| Honduras | 0.26 (0.00-2.63) | 25 (1-77) | 12 (0-106) | 0 (0-0) | 36 (1-174) | 34 | 4.72 |
| Venezuela | 0.63 (0.00-6.31) | 7 (1-17) | 10 (0-70) | 0 (0-0) | 17 (1-82) | 118 | 4.54 |
| French Guiana | 0.00 (0.00-0.00) | 6 (1-14) | 5 (0-44) | 0 (0-0) | 11 (1-49) | 0 | 4.53 |
| Bolivia | 0.26 (0.00-2.61) | 3 (0-15) | 1 (0-10) | 0 (0-0) | 5 (0-28) | 74 | 5.60 |
| Guyana | 0.05 (0.00-0.53) | 2 (0-5) | 0 (0-1) | 0 (0-0) | 2 (0-6) | 58 | 2.83 |
| Suriname | 0.01 (0.00-0.05) | 0 (0-1) | 0 (0-1) | 0 (0-0) | 0 (0-2) | 12 | 4.12 |
| Belize^5^ | 0.000 | 0 (0-0) | 0 (0-0) | 0 (0-0) | 0 (0-0) | 3 | - |
| Panama^5^ | 0.032 | 0 (0-0) | 0 (0-0) | 0 (0-0) | 0 (0-0) | 5 | - |
|  |  |  |  |  |  |  |  |
| **CSE Asia region_1_** |  |  |  |  |  |  |  |
| India | 60.28 (0.00-602.84) | 9,964 (5,986-13,818) | 51,129 (19,268-87,477) | 40,374 (1,934-106,933) | 101,527 (31,010-186,986) | 10,650 | 1.53 |
| Myanmar | 0.00 (0.00-0.00) | 1,075 (482-1,951) | 11,920 (7,162-17,026) | 13,110 (2,689-29,303) | 26,105 (14,123-41,132) | 4,209 | 1.03 |
| Indonesia | 8.61 (0.00-86.13) | 1,812 (1,089-2,503) | 6,780 (3,300-11,057) | 3,740 (979-9,757) | 12,341 (6,218-20,899) | 2,518 | 1.18 |
| Bangladesh | 4.90 (0.00-48.99) | 301 (1-1,034) | 3,452 (440-7,860) | 5,617 (0-19,601) | 9,375 (1,216-20,636) | 2,975 | 2.07 |
| Philippines | 2.09 (0.00-20.90) | 602 (157-1,183) | 2,784 (376-7,031) | 2,343 (0-11,737) | 5,732 (695-16,834) | 124 | 2.81 |
| Thailand | 3.14 (0.00-31.39) | 448 (257-722) | 2,094 (321-3,926) | 1,396 (2-5,611) | 3,941 (650-9,716) | 257 | 2.29 |
| Viet Nam | 5.48 (0.00-54.82) | 634 (344-948) | 2,474 (619-6,315) | 607 (0-2,362) | 3,721 (1,289-9,438) | 70 | 2.18 |
| Pakistan | 6.89 (0.00-68.89) | 671 (191-1,294) | 1,755 (73-6,331) | 831 (0-7,350) | 3,264 (378-14,101) | 1,499 | 4.19 |
| Cambodia | 0.26 (0.00-2.60) | 306 (96-560) | 1,755 (92-4,115) | 659 (0-4,375) | 2,720 (223-7,471) | 262 | 2.66 |
| Lao People's Democratic Republic | 0.00 (0.00-0.04) | 120 (40-225) | 933 (295-1,739) | 673 (1-2,252) | 1,727 (485-3,765) | 22 | 1.90 |
| Malaysia | 1.65 (0.00-16.48) | 146 (38-278) | 892 (200-1,674) | 686 (4-1,992) | 1,726 (424-3,455) | 15 | 1.75 |
| Papua New Guinea | 0.00 (0.00-0.00) | 105 (59-153) | 795 (380 - 1,195) | 721 (109 - 1,902) | 1,622 (725 - 2,941) | 1,508 | 1.37 |
| China | 2.04 (0.00-20.42) | 383 (78-720) | 806 (94-2,952) | 218 (0-1,689) | 1,410 (214-5,559) | 100 | 3.78 |
| Afghanistan | 1.25 (0.00-12.55) | 145 (58-243) | 434 (11-1,335) | 139 (0-1,173) | 719 (109-2,256) | 586 | 2.97 |
| Sri Lanka | 0.78 (0.00-7.77) | 51 (0-128) | 211 (0-848) | 72 (0-747) | 335 (2-1,324) | 3 | 3.93 |
| Solomon islands | 0.00 (0.00-0.00) | 9 (0-32) | 92 (3-211) | 166 (0-550) | 267 (7-638) | 106 | 2.36 |
| Timor-Leste | 0.00 (0.00-0.00) | 21 (2-40) | 125 (0-306) | 106 (0-561) | 252 (15-708) | 529 | 2.75 |
| Bhutan | 0.05 (0.00-0.46) | 22 (0-54) | 56 (0-375) | 87 (0-978) | 165 (1-1,022) | 16 | 6.19 |
| Vanuatu | 0.00 (0.00-0.00) | 8 (3-16) | 21 (0-73) | 5 (0-37) | 34 (4-118) | 30 | 3.37 |
| Nepal | 0.62 (0.00-6.22) | 11 (0-88) | 4 (0-14) | 0 (0-0) | 16 (0-110) | 31 | 6.92 |
| Iran^5^ | 0.612 | 0 (0-0) | 0 (0-0) | 0 (0-0) | 0 (0-0) | 18 | - |
| Kyrgyzstan^5^ | 0.000 | 0 (0-0) | 0 (0-0) | 0 (0-0) | 0 (0-0) | 0 | - |
| Tajikistan^5^ | 0.006 | 0 (0-0) | 0 (0-0) | 0 (0-0) | 0 (0-0) | 2 | - |
|  |  |  |  |  |  |  |  |

1. The regional groupings are illustrated in Figure 2A of the main text.

2. Estimated *P. falciparum* cases in areas of stable transmission are shown disaggregated into three policy relevant classes of endemicity, defined previously [7].

3. These figures are estimates of malaria cases made by the World Health Organization and presented in the World Malaria Report 2008 [2].

4. Ratio of the width of the 95% confidence intervals to the point estimate for total clinical cases per country.

5. Case numbers used directly from those countries considered to have reliable reporting systems.

| **Table S1.2.** Estimated *P. falciparum* clinical cases (thousands) by global regions in 2007 | | | | | |
| --- | --- | --- | --- | --- | --- |
|  | **Unstable risk** | ***Pf*PR_2-10_ ≤5%_2_** | ***Pf*PR_2-10_ >5-<40%_2_** | ***Pf*PR_2-10_ ≥40%_2_** | **Total** |
|  |  |  |  |  |  |
| **MAP regions_1_** |  |  |  |  |  |
| Americas region | 5.45 (0.00-54.50) | 1,430 (965-2,096) | 1,516 (116-4,490) | 96 (0-1,078,545) | 3,048 (1,169-6,759) |
| Africa+ region | 1.89 (0.00-18.90) | 6,669 (4,939-8,425) | 82,217 (70,526-96,718) | 181,999 (145,203-220,361,420) | 270,887 (241,127-300,578) |
| CSE Asia region | 98.05 (0.00-980.50) | 16,835 (12,865-20,589) | 88,512 (46,711-125,667) | 71,553 (20,570-152,400,881) | 176,998 (89,206-270,563) |
|  |  |  |  |  |  |
| **WHO regions** |  |  |  |  |  |
| AFRO | 0.56 (0.00-5.69) | 5,382 (3,667-7,032) | 75,972 (63,484-90,003) | 179,639 (142,011-216,737,879) | 260,994 (231,929-288,387) |
| EMRO | 9.48 (0.00-94.80) | 2,104 (1,474-2,906) | 8,433 (4,881-15,676) | 3,330 (885-11,690,956) | 13,876 (8,203-26,029) |
| AMRO | 5.46 (0.00-54.6) | 1,430 (965-2,096) | 1,516 (116-4,490) | 96 (0-1,078,545) | 3,048 (1,169-6,759) |
| EURO | 0.00 (0.00-0.00) | - | - | - | - |
| SEARO | 78.38 (0.00-783.80) | 13,705 (9,615-17,312) | 75,770 (37,533-110,402) | 64,504 (16,617-145,812,209) | 154,057 (68,829-243,484) |
| WPRO | 11.53 (0.00-115.30) | 2,314 (1,473-3,084) | 10,553 (4,319-17,509) | 6,079 (1,502-14,855,005) | 18,958 (7,764-31,529) |
|  |  |  |  |  |  |
| **Global** | 105.40 (0.00-1,053.95) | 24,935 (20,694-29,800) | 172,245 (121,815-206,883) | 253,648 (183,633-343,495,980) | 450,933 (348,764-553,269) |
|  |  |  |  |  |  |

1. The regional groupings are illustrated in Figure 2A of the main text.

2. Estimated *P. falciparum* cases in areas of stable transmission are shown disaggregated into three policy relevant classes of endemicity, defined previously [7].

#### References

1. Guerra CA, Gikandi PW, Tatem AJ, Noor AM, Smith DL, et al. (2008) The limits and intensity of *Plasmodium falciparum* transmission: implications for malaria control and elimination worldwide. PLoS Med 5: e38.

2. W.H.O. (2008) World malaria report 2008. WHO/HTM/GMP/2008.1. Geneva: World Health Organization. 215 p.

3. W.H.O. (2009) World malaria report 2009. Geneva: World Health Organization. 202 p.

4. Cibulskis RE, Bell D, Christophel EM, Hii J, Delacollette C, et al. (2007) Estimating trends in the burden of malaria at country level. Am J Trop Med Hyg 77: 133-137.

5. Rowe AK, Rowe SY, Snow RW, Korenromp EL, Schellenberg JRA, et al. (2006) The burden of malaria mortality among African children in the year 2000. Int J Epidemiol 35: 691-704.

6. Craig MH, Snow RW, le Sueur D (1999) A climate-based distribution model of malaria transmission in sub-Saharan Africa. Parasitol Today 15: 105-111.

7. Hay SI, Guerra CA, Gething PW, Patil AP, Tatem AJ, et al. (2009) A world malaria map: *Plasmodium falciparum* endemicity in 2007. PLoS Med 6: e1000048.
